# An Intelligent Man-Machine Interface—Multi-Robot Control Adapted for Task Engagement Based on Single-Trial Detectability of P300

**DOI:** 10.3389/fnhum.2016.00291

**Published:** 2016-06-21

**Authors:** Elsa A. Kirchner, Su K. Kim, Marc Tabie, Hendrik Wöhrle, Michael Maurus, Frank Kirchner

**Affiliations:** ^1^Research Group Robotics, Mathematic and Computer Science, University of BremenBremen, Germany; ^2^Robotics Innovation Center (RIC), German Research Center for Artificial Intelligence (DFKI GmbH)Bremen, Germany

**Keywords:** EEG, P300, machine learning, space robotics, teleoperation, task load, man-machine interaction, embedded brain reading

## Abstract

Advanced man-machine interfaces (MMIs) are being developed for teleoperating robots at remote and hardly accessible places. Such MMIs make use of a virtual environment and can therefore make the operator immerse him-/herself into the environment of the robot. In this paper, we present our developed MMI for multi-robot control. Our MMI can adapt to changes in task load and task engagement online. Applying our approach of embedded Brain Reading we improve user support and efficiency of interaction. The level of task engagement was inferred from the single-trial detectability of P300-related brain activity that was naturally evoked during interaction. With our approach no secondary task is needed to measure task load. It is based on research results on the single-stimulus paradigm, distribution of brain resources and its effect on the P300 event-related component. It further considers effects of the modulation caused by a delayed reaction time on the P300 component evoked by complex responses to task-relevant messages. We prove our concept using single-trial based machine learning analysis, analysis of averaged event-related potentials and behavioral analysis. As main results we show (1) a significant improvement of runtime needed to perform the interaction tasks compared to a setting in which all subjects could easily perform the tasks. We show that (2) the single-trial detectability of the event-related potential P300 can be used to measure the changes in task load and task engagement during complex interaction while also being sensitive to the level of experience of the operator and (3) can be used to adapt the MMI individually to the different needs of users without increasing total workload. Our online adaptation of the proposed MMI is based on a continuous supervision of the operator's cognitive resources by means of embedded Brain Reading. Operators with different qualifications or capabilities receive only as many tasks as they can perform to avoid mental overload as well as mental underload.

## 1. Introduction

Human-robot interaction with semi-autonomous robots has to be improved to be safe and intuitive. This can be achieved by (1) building robots with advanced “on-board” solutions that support natural interaction behavior between human and robot (Kirchner et al., 2015) and (2) by developing intelligent man-machine interfaces (MMIs). Especially in cases of tele-operating robots at remote places the MMI has to be easy, intuitive and comfortable.

Usually only experienced people are chosen to remotely operate robotic systems (Cornellă et al., 2012), since their performance is robust. During remote control of several robots in a complex mission, task load and task engagement change tremendously over time, which can lead to mental over- or underload as well as fatigue. Therefore, an online-adaptable MMI can be applied to act on these changes. For this, reliable measures for online changes in the human's state must be detected (Allanson and Fairclough, 2004). Such realtime indicators have to consider theories about brain capacity and resources (Kahneman, 1973; Wickens, 1984, 1992, 2008), which propose that brain resources are limited and must be shared between tasks. Comprehensive work showed that certain patterns in the electroencephalogram (EEG), e.g., the amplitude of the event-related potential (ERP) P300 (Prinzel et al., 2003), or ratios of EEG power bands like alpha, beta or theta bands (Pope et al., 1995), can be used to measure the processing capability of the brain, mental workload and task demands. In earlier work from Pope et al. (1995) it is shown that an EEG-based index of user engagement and arousal could indeed be used to, i.e., adapt the level of system automation in response to changes in mental workload demands. It was found that especially the P300 is a reliable measure for changes in task load (Kok, 2001; Prinzel et al., 2003). Earlier work that examined the P300 in response to primary and secondary task demands showed that an increase in demands on the primary task resulted in fewer resources for the secondary task accompanied by a smaller P300 amplitude (Isreal et al., 1980). Many studies make use of the dual-task design (Isreal et al., 1980; Prinzel et al., 2003) to detect an increase in workload or task load in the primary task by analyzing the P300 amplitude evoked by the secondary task, e.g., listening to auditory stimuli presented in an oddball fashion (Prinzel et al., 2003) or P300 that is evoked by ignored probes (Kramer et al., 1995).

With the focus on online user state detection based on the analysis of brain activity, which is naturally evoked during human-machine interaction and deeply embedded into the systems control, embedded Brain Reading (eBR) was developed (Kirchner and Drechsler, 2013; Kirchner, 2014, 2015). The main focus of embedded Brain Reading is to passively infer on the human's intention to implicitly improve interfaces like an exoskeleton which is used for explicit interaction, such that the intended interaction or behavior can be supported best (Folgheraiter et al., 2012; Kirchner et al., 2013a,b, 2014). However, embedded Brain Reading can also be applied to passively infer on the users' neurophysiological state, such as their current workload or task load, to adapt an interface implicitly in such a way that the user is neither stressed nor bored (Kirchner et al., 2010, 2013b; Wöhrle and Kirchner, 2014a) which would both have negative impact on human-robot interaction. We already showed that eBR can utilize P300-related activity to infer, whether subjects recognize and will respond to important task messages, which were presented interleaved with task-irrelevant messages in an oddball fashion, while performing a complex interaction task like playing a labyrinth game (Kirchner et al., 2013b). In a later work we showed that eBR can indeed be applied to improve interaction in an application scenario in which subjects had to respond to warnings interleaved with task-irrelevant status messages while remotely controlling a robotic arm via an exoskeleton (Wöhrle and Kirchner, 2014a). In both cases, the information about the operator's capability of recognizing task-relevant warnings was used to adapt the developed MMI with respect to the timing of repetitions of task messages. To this end, the MMI was adapted before the operator would respond to the task message. In our previous work, subjects had to perform two tasks: controlling a machine and responding to task-relevant warnings. Thus, we did not make use of the primary and secondary task design just for the purpose of measuring task load on the user. The second task was indeed required to be performed by the user with the goal to estimate an operator's capability to perform two tasks at the same time. We also believe that even when using ignored probes to measure load on the user, i.e., workload (Kramer et al., 1995), any extra stimulation which is only added for the purpose of measuring load on the user will likely disturb the operator in a complex and demanding interaction task. Instead, we used the single-trial detectability of the naturally evoked P300 components in case that rare task-relevant stimuli were presented (i.e., warnings that anyway requested responses of the operator) and had to be answered as index of load, here, task load and task engagement. However, in many real world applications the occurrence of task-relevant target stimuli is likely not interleaved consistently with task-irrelevant stimuli as it was implemented in the previous studies by using the oddball design. Thus, it is of interest to investigate whether single-target stimuli successfully and reliably evoke P300 ERP components during human-machine interaction, as suggested by comprehensive work performed under controlled conditions of the single-stimulus paradigm (Mertens and Polich, 1997; Polich and Margala, 1997). Polich and Margala (1997) for example showed, that single-target stimuli evoke P300 components with similar characteristics as target stimuli presented in an oddball fashion as long as the probability and the inter target interval (ITI) were kept the same.

One research interest of the current work is therefore to investigate whether P300 ERP components are reliably evoked under application conditions in case of a single-stimulus presentation that was naturally embedded into a human-machine interaction task. We further investigate whether eBR can be used to adapt the frequency of task messages that are presented to the user by an MMI instead of modulating task repetitions as in a former work (Kirchner et al., 2013b; Wöhrle and Kirchner, 2014a). The adaptation of the MMI should again be performed online. However, the proposed MMI is designed for multi-robot control. Hence, an adaptation of the MMI with respect to the inferred task load and the users current task engagement in preceding, still ongoing, tasks for other robots can be investigated. Again, task engagement or task load was inferred from P300-related ERP activity that is naturally evoked during interaction. Both a high task load and a high task engagement to a preceding task were expected to reduce the amplitude of P300-related activity evoked by a new task message. In the presented work, subjects performed only one type of task: controlling different robots with respect to different requested tasks. Hence, we break down dual-task execution into sequential and timely overlapping task execution to investigate the influence of task load and task engagement between subsequent tasks. We again show that it is not necessary to artificially add an extra task or probe, like in the dual task or ignored-probe design, to evoke P300-related activity for measuring task load and task engagement. Instead we directly infer the task load and task engagement of the operator from the P300-activity evoked by task messages.

Hence, our approach matches natural requirements on the user during robot control since it avoids to add potentially disturbing stimuli, like auditory stimuli, just for the goal to measure and adapt for task load.

**Figure 1:**
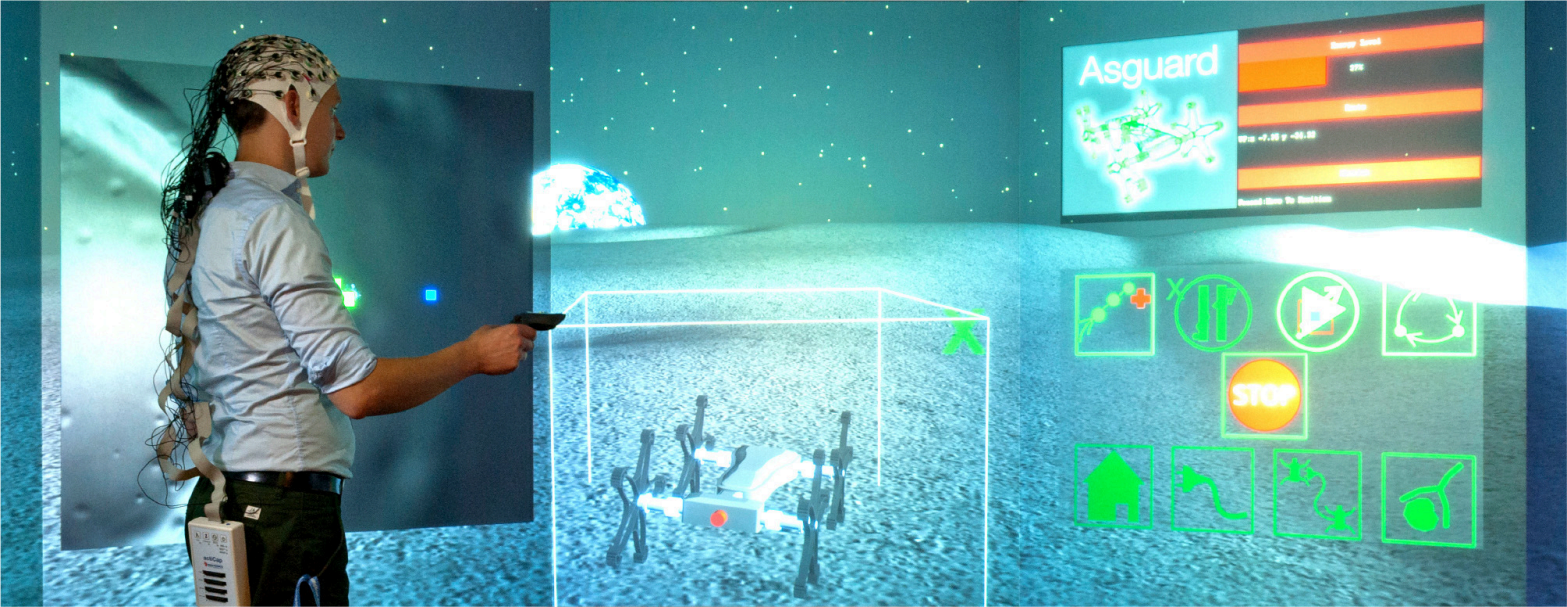
**Immersive virtual 3D multi-robot control using a CAVE supported by embedded Brain Reading (eBR)**.

We further present and describe the developed MMI, which makes use of a virtual control environment, i.e., a Cave Automatic Virtual Environment (CAVE) (Figure 1). This MMI can be adapted based on the changes in task engagement of the user measured by EEG, i.e., P300-related ERP activity. While the presentation of each task-relevant message was expected to evoke a P300 we further assumed that the amplitude of a single-trial P300 evoked by a new task message is reduced in case that the user is still involved in executing a previous task. This is due to the fact that mental resources are still bound to the previous task. The more frequently such task conflicts occurred the stronger we expected a reduction in averaged P300 peak amplitude. We further assumed that the expected changes in P300 amplitude were mainly caused by effects like task engagement or task load but not by target probability, since the inter-stimulus interval (ISI) between stimuli was very long. Polich (1990) showed by means of an auditory discrimination task that the target probability has no effect on P300 amplitude in case of longer ISIs, i.e., ISIs longer than 6–8 s (Polich, 2007). For longer ISIs, the probability effect (Tueting et al., 1970; Duncan-Johnson and Donchin, 1977) is missing since brain resources can be redirected fast enough to process a new target stimulus.

It is important to state that in the present work the level of task load and task engagement as well as the occurrence of task conflicts may strongly depend on different factors, e.g., the general capability of the user in controlling the robots, fatigue levels or secondary requirements on attention that are not related to the main task, i.e., distractions of any kind that may occur while the operator was controlling the robots. While the concept of workload is distinct from the concept of multiple resource theory (Wickens, 2008), both concepts do overlap in real world applications and it is not always clear what contributes most. Moreover, additional mechanisms like confusion, cooperation between task elements like ongoing task engagement to the preceding task and unwanted diversion of attention influence the allocation of brain resources (Wickens, 2008). Additionally, as known from educational research, changes in the motivational state influence perception of workload, task complexity and cognitive strategies (Kyndt et al., 2011). Real world applications are therefore not a good paradigm to decouple components and dimensions of influencing parameters, but they can be used as a test case on whether certain measures can be used to predict the general state and capacities of a subject. Since the goal of our study was to measure the current task engagement or task load of an operator and to use this measure to adapt an MMI continuously to avoid an overall state of overload, we took measures to avoid excessive workload.

In summary, the scope of this study was to artificially evoke task conflicts to (I) not only show that P300-related activity was naturally evoked when task messages were presented, but also that it was indeed modulated by generally high demands on the operator and by task engagement to previous tasks and (II) that the detectability of P300-related activity could be used to adapt an MMI with regards to task engagement and therefore enabling a kind of steady-state task involvement. This should result in higher subjective contentment and high overall task performance.

The paper is structured as follows. In Section 2 we describe the experimental setting, i.e., the developed MMI, the kind of human-machine interaction task which can be performed and the interaction tasks that the subjects had to solve, the experiments that were performed for this work, and data recording procedure. We further describe our research goals and hypotheses in more detail and describe the performed data processing and analysis. In Section 3 we describe our results with respect to behavioral, machine learning and ERP average analysis. Finally in Section 4 we will discuss the outcome of our work and its relevance for the improvement of MMIs for multi-robot control.

## 2. Materials and methods

### 2.1. Experimental design

**Figure 2:**
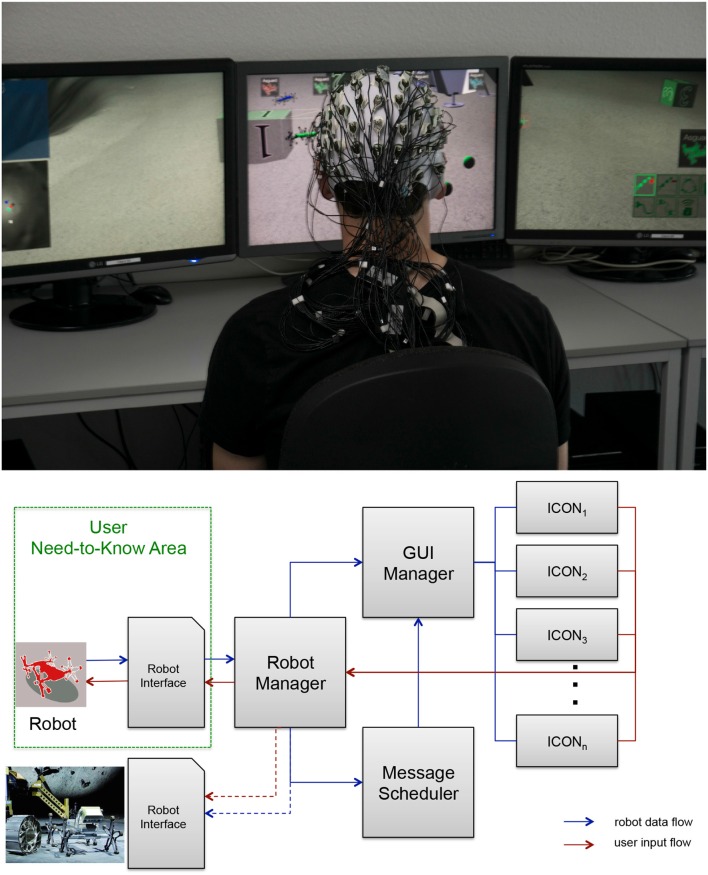
**Experimental setup**. **Upper part**: Virtual multi-robot control in 2D using a multi-PC system supported by embedded Brain Reading (eBR). **Lower part**: Interaction is controlled by different software managers and schedulers. Widget-based icons are used to display information about the robots, messages for the user and to select robot commands. The user “Need-to-Know Area” is the part of the system visible to the user. The robot interface with connections to the real robots (depicted by dotted lines) is not yet implemented.

We developed an experimental setup in which a subject can control several simulated robots. For this, we designed a virtual environment using the in-house developed software “Machina Arte Robotum Simulans” (MARS) (Rommerman et al., 2009; DFKI - RIC, 2015), which can be run as a 3D environment in, e.g., a CAVE (see Figure 1), as a 2D environment on a standard personal computer and monitors or a multi-screen system (see Figure 2). In both environments the operator can use different input devices to control the robot, e.g., a 3D mouse, a wand, an exoskeleton or an eye tracking device. In the future, the developed virtual 3D environment will be used to control real robots. To allow this, we use a physical simulation with close to realistic physical simulations of the real robots developed at our institute. In this work a 2D multi-screen system was used as the environment and a wand was used as the interface to control the simulated robots in the simulated environment. The used wand is a hardware device and functions in a 3D environment similar to a mouse in a 2D environment. It is tracked in 3D space using an ultrasound-based tracking system combined with an IMU and has five buttons as well as a pressure-sensitive joystick as input options. We used the inertial-ultrasonic hybrid tracking device InterSense IS-900 (Thales Visionix, Inc., Billerica, USA) in our experiments.

#### 2.1.1. Human-robot-interaction

**Figure 3:**
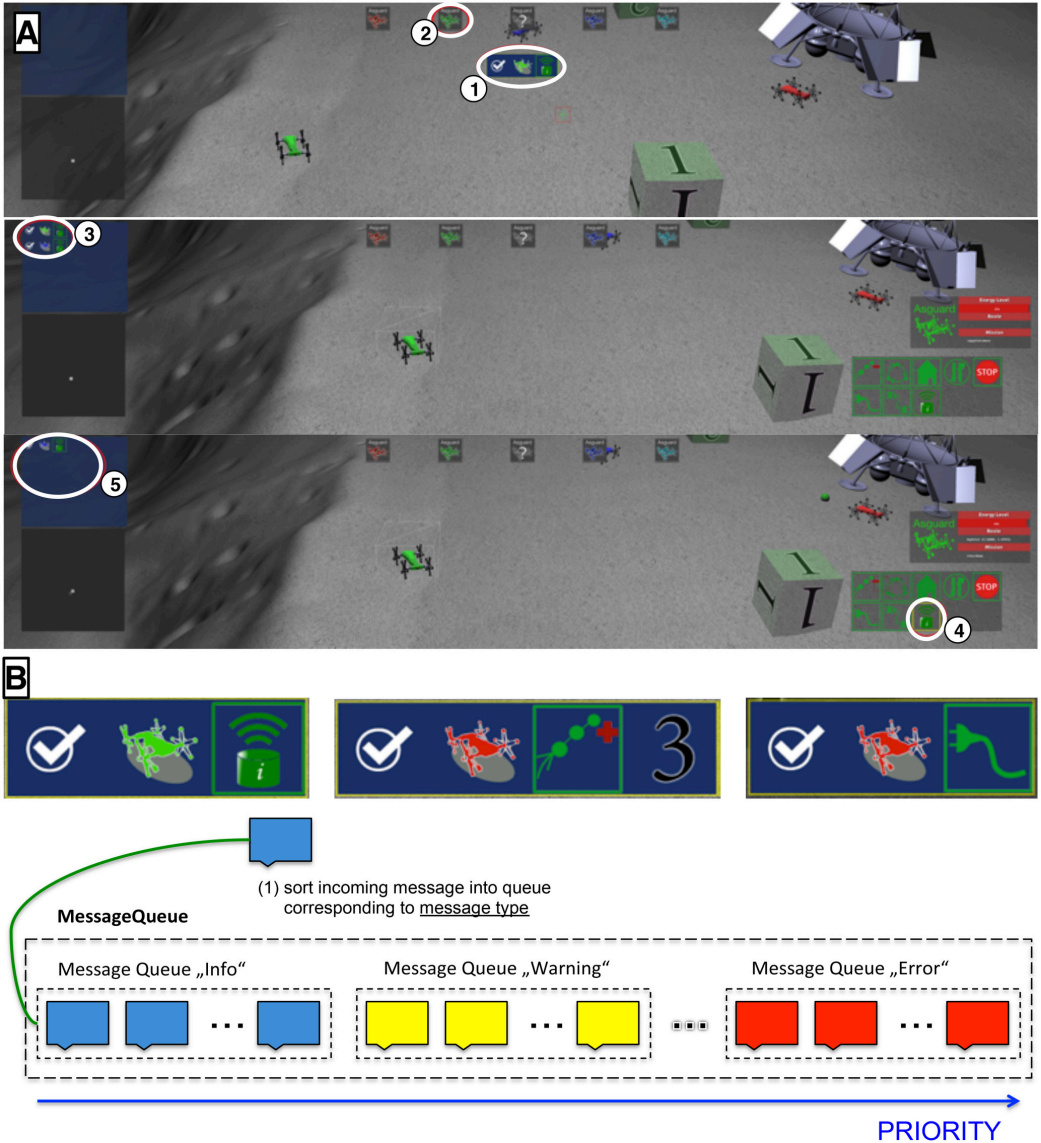
**Description of experimental setting and tasks performed by the operator. A (top)** initial state, a task message was shown to the operator (1). The message contained information about the type of the task (e.g., send a message) and the corresponding robot (e.g., the green robot). The subjects had to confirm the task by clicking on a response button (2). **A (middle)** after the task was confirmed, it was shown in the task manager (3). **A (bottom)** when the green robot was selected, a menu with all possible control commands was shown. In this example, the mission could be accomplished by clicking on the send-message button of the control menu (4). When a task was accomplished, it was removed from the task manager (5). **(B)** The scenario contained three possible tasks, which were depicted by an intuitive symbol. All tasks were related to a specific robot, encoded by a colored symbol, see the following examples. **B (top left)** send a message with the green robot. **B (top middle)** send the red robot to waypoint 3. **B (top right)** recharge the red robot. Different robots (encoded by color) and different task messages were randomly combined. **B (bottom)** messages are sorted in order as they are presented. Some messages (repetitions of tasks) get a higher priority and will be presented earlier.

In general, the task of the operator in the multi-robot control environment (see Figure 2) was to supervise all robots and to assign new tasks to individual robots as indicated by messages presented to the user on the screen (see Figures 3A,B upper part for examples of different messages). Individual robots were labeled with different colors. Task messages were presented as icon based widgets supporting fast recognition by the operator. The operator used the interface to select a robot he or she wanted to control by either selecting the robot directly or by selecting the robot's icon in the upper part of the middle screen (see Figure 3A: 2). Moreover, information about the chosen system was presented to the operator on the right screen via an icon based information panel. Information such as the robot's name, its energy level, its current task as well as robot control commands were presented here (see Figure 3A: middle picture lower right corner). On the left monitor, tasks for the operator were listed as soon as the operator confirmed that he/she had seen the message by clicking on the appropriate robot icon on the monitor in the middle. By selecting the robot's icon with a double click, the virtual camera was additionally moved such that the chosen robot was in the focus of the operator. After selecting a robot, the operator can issue a task by clicking the corresponding robot control command icon. (see Figure 3A: 4). In case that an operator was not sure or did not recognize the robot to whom a task was assigned, he or she could select an unknown icon displaying a gray robot with a question mark (see Figure 3A). After clicking the unknown icon, all the missed tasks were displayed in the task list on the left screen. However, in the experiments presented here this gray robot button was disabled to force the subjects to focus on the task messages as much as possible. In case that a user did not recognize the task message correctly she or he had to wait for the automatic repetition of the task message.

#### 2.1.2. Interaction tasks

As mentioned in Section 2.1.1 the operator had to fulfill different tasks with the robots. Within the experiment there were three kinds of tasks with varying complexity:

- **Send message** The task with the lowest complexity is sending a message. This task can be solved by selecting the corresponding robot and clicking on the send-message icon within the robots control elements (see Figure 3A bottom number 4). An example of such a message for the green robot can be seen in the upper left part of Figure 3B.
- **Go to landmark** The task with a medium complexity is the navigation task. Within the experiment there are five different landmarks (for example see the cube labeled with 1 in Figure 3A). The goal of this task is to navigate the robot to one of these landmarks. Therefore the operator needs to select the robot and afterwards plan the path by creating waypoints. Waypoints will be put at the position of the cursor, when clicking a specific button. The robot will consecutively travel from waypoint to waypoint on straight lines. When the robot reaches the landmark the task is fulfilled. An example of such a message for the red robot with target position 3 can be seen in the upper middle part of Figure 3B.
- **Recharge robot** The most complex task is recharging the robot. Again the correct robot needs to be selected first. Afterwards the operator has to plan a path to the lander (see in the top right corner of Figure 3A). The path planning is realized as explained above in the “Go to landmark” section. After reaching the lander the robot needs to be selected again and the recharge icon from the robot's control elements needs to be activated by clicking on it. This task is more complex than the “Go to landmark” task due to a gap in between the two stages of the task and therefore the operator must track the robot's state. The operator may also forget to click the recharge icon after the robot reached the lander. An example of such a message for the red robot can be seen in the upper right part of Figure 3B.

All tasks were pseudo-randomly chosen, such that no more than one task at a time was assigned per robot. When creating a new “Go to Landmark” task for a specific robot the robot's distance to the landmarks will be computed first. In order to solve the task the robot has to be in a specific radius around the chosen landmark. If the robot is already within the specific radius the new task would directly be solved when the robot is selected. In such a case the target landmark will be chosen among the other landmarks. Further, there was an automated mechanism which generated a “Recharge Robot” task in case that the energy level of a robot dropped bellow a certain value. This was necessary to ensure that a robot would remain fully functional. If a robot runs out of energy it would get stuck at its position and no more tasks could be solved by this robot.

When a message was presented requesting interaction the first response of the user like selecting the correct robot was counted as correct behavior. The message was not repeated. On the other hand, a predefined response time (in our experiments 13 s) and a predefined ISI was set for the operator. The predefined ISI was important for our experiments and research questions as will be explained in Section 2.4. Task messages were put into a message queue. To avoid unfair scheduling due to different urgency of information pending messages may change their priority over time (see Figure 3B lower part). So far it is implemented that a message is repeated as a warning in case that a complex task with longer duration is started, i.e., a robot is sent to a landmark, but does not arrive after a certain amount of time. Since the robot might have got stuck the warning is repeated with higher priority. To give the user an overview on initiated but still running tasks, they were visualized in a icon panel in the upper left corner of the left monitor in the order as they appeared with the newest depicted on the top (see Figure 3A: 5). As soon as a task was fulfilled the task message was removed.

### 2.2. Performed experiments

Six subjects participated in the study. All subjects were male with normal or corrected to normal vision and aged between 20 and 38 years (mean: 28.74, SD: 6.92). All subjects were intensively trained in the scenario on a different day to get used to the tasks, i.e., to control the robots by using the developed MMI. On the same day of the study just before data recording subjects were asked to get comfortable with the scenario. The study consisted of 6 runs, performed in the same order. In each run, subjects had to complete 30 tasks. The response behavior was supervised and logged by the message scheduler (see Figure 2 lower part).

In case no response was detected within 13 s after presentation of a task message, the same task message was again attached to the message queue. Since the queue is implemented as a FIFO (first in first out), the message is repeated after presentation of all other messages within the queue.

Task messages (Figure 3 top illustration and Figure 3B) were presented for 1.1 s. The duration of presentation was determined by empirical tests with a different group of 4 subjects. The goal was to keep the duration of message presentation as short as possible to allow the evaluation of event-related activity in the EEG while ensuring that subjects were able to recognize and understand the presented messages.

#### 2.2.1. Adaptation of the inter-stimulus interval (ISI)

Between the 6 runs experimental conditions were varied with respect to the ISI (**Table 2.1**: EEG data). For runs 1 to 4 ISIs were fixed. We used two different ISIs: a long ISI (25 s) in runs 1 and 2 and a short ISI (15 s) in runs 3 and 4. In both cases an additional random jitter of ±5 s was added. Appropriate time intervals for long and short ISIs were empirically determined beforehand by tests with 4 subjects that were not involved in this study. The time interval for the short ISI was chosen such that the overall workload or overall task load caused by the message frequency was not too high. We were successful in empirically determining an appropriate time interval for short ISIs as supported by results of the evaluation of the NASA Task Load Index questionnaire (see Section 3.1.3). The time interval for the long ISI was empirically chosen to be clearly higher in the subjective perception of the 4 test subjects. A very low ISI could not be chosen, since we experienced that subjects easily gave up the run in cases of very short ISIs, i.e., with a duration of 5 s or even with a duration of 10 s. Further, no P300 was evoked under extremely stressful circumstances, as in runs with an ISI of 5 s. Moreover, to train the classifier qualitatively good training examples were required. And finally, we had to limit the number of runs and thus total experiment time to avoid overstraining the subjects.

For runs 5 and 6 the ISI was adapted online with respect to detectability of the P300 and related ERP activity. For the online detection of single-trial ERP activity a classifier was trained on examples from either runs 1 and 2 (for application in run 5) or on examples from runs 3 and 4 (for application in run 6) (see Section 2.8 for more details). Adaptation in runs 5 and 6 of the ISI was increased gradually (up to a maximum of 35 s in steps of 5 s) in case that an expected P300 was not detected two times in a row after a new task message or was decreased stepwise (down to a minimum of 5 s in steps of 5 s) in case that an expected P300 was detected two times in a row. For both adapted runs the ISI was preset to 25 s. We always startet with the fixed ISI condition with an ISI of 25 s in runs 1 and 2 to allow subjects to get comfortable with the control task. This was done since long training sessions just before the experimental session were not possible since they would have increased the total experiment time to an unacceptable long duration. For our experimental setting it was more important to record all runs in the same session to avoid between-session effects on the shape of the ERPs as well as the single-trial classification performance. Although subjects were intensively trained, they needed to readapt to the control of the robots, since the control task was very complex. Next, in runs 3 and 4 training data was recorded under the fixed ISI condition. We did not perform a run with adapted ISI right after the recording of training data with ISI 25 to keep both runs with adapted ISI close together and thus condition of the subjects similar. Further, interleaving runs with fixed and adapted ISIs were not performed, since this might have had an influence on the motivation of the subject during the recording of training data after a run with adapted ISI.

#### 2.2.2. Ethics statement

The study has been conducted in accordance with the Declaration of Helsinki and approved with written consent by the ethics committee of the University of Bremen. Subjects have given informed and written consent to participate.

### 2.3. Recorded data

During each executed run EEG was recorded with 64 electrodes referenced against electrode FCz. An actiCap system (Brain Products GmbH, Munich, Germany) arranged as an extended 10–20 system was used for recording. Electrode impedance was kept below 5 kΩ. EEG signals were sampled at 5 kHz, amplified by two 32 channel BrainAmp DC amplifiers (Brain Products GmbH, Munich, Germany) and filtered with a low cutoff of 0.1 Hz and high cutoff of 1 kHz.

### 2.4. Research goals & hypotheses

The presented work addresses two different research goals with specific subgoals. (I) We want to show that a P300-related activity is naturally evoked when task messages are presented and recognized. (Ia) We investigate whether the evoked P300 is modulated by factors like demands on the operator or the operator's task engagement to previous tasks. (II) We want to show that single-trial detection of P300-related activity can be used to adapt the interaction with respect to the task engagement of the operator. (IIa) In particular, we investigate whether an individual balanced task involvement of the operator can be achieved by adaptation of the ISI resulting in a higher subjective contentment of the operator and in an individually optimized overall task performance.

By means of data recorded in runs 1–4 we investigated research goal (I). We artificially modulated the current task engagement (on the previous task) by presenting a new task. This was achieved by modulating the time interval between both consecutive tasks: long ISIs of 25 seconds in runs 1 and 2; short ISIs of 15 s in runs 3 and 4. Changes in P300 characteristics were investigated by averaged ERP analysis and machine learning methods. To support the usage of single-trial P300 detection we had to assure that the detection performance is adequately high and not too strongly influenced by ISI per se such that for very short ISIs possibly no P300 would be detectable in single-trial. For this, an *offline* machine learning analysis was performed first with training and test on runs with the same ISI. These results were used as a baseline for other experiments. This condition was called “baseline” condition. Using this analysis, we investigated whether P300-related activity is detectable in single-trial under application conditions and for different ISIs as well as how strongly different ISIs would influence classification performance.

Further, we investigated the effect of classifier transfer between runs with different ISIs. More precisely, a transfer of classifier between training runs (runs 1 and 2 or runs 3 and 4) and test runs (runs 5 and 6 with adapted ISI) was applied. This condition was called “transfer” condition. This offline analysis was relevant because under the *online* condition the classifier was transferred between different ISI conditions. Different ISIs were caused by the adaptation of the ISI under the *online* condition. Results allow to estimate the sensibility of the classifier for changes in ISI.

To achieve research goal (II) we adapted the developed MMI with respect to the current task engagement of the user to previous tasks when a new task was presented in runs 5 and 6 (**Table 2.3**: online stCL). Current task engagement was measured by the *online* single-trial classification of P300-related activity evoked by recognized target stimuli, i.e., task messages: (1) task engagement to a previous task was expected to be high in case that the P300-related activity was weakly evoked by a new task and thus not detected by a classifier, (2) task engagement to a previous task was expected to be low in case that P300-related activity was more strongly expressed and thus detected by a classifier. Note that in the online case each EEG trial after a presented first task message was classified, thus in case the operator completely missed a task message no P300 was expected to be evoked and could therefore not be detected. Hence, our approach did not only account for reduced P300 activity but also for missed P300 in case of missed target events.

To prove that the interaction of the user was improved by online adaptation of the ISI, we analyzed the total runtime, median reaction time and number of late responses and missed messages. We expected a reduction in total runtime by online adaptation of the ISI compared to the case of a fixed long ISI (ISI-25; runs 1 and 2). We did not expect a significant difference to be found for reaction times, since our approach would avoid user overload and responses were rather complex (see Section 2.1). However, we expected some late responses and missed messages in cases that the user was strongly involved in ongoing tasks when a new task was presented.

Our approach of online adaptation of the ISI allows to adapt an MMI with respect to the current task engagement or task load, improves user performance by equalizing the level of task engagement over all tasks and by selectively avoiding task overload. To further support this, we investigated the effect of an online adaptation of the ISI on averaged P300-related activity, i.e., we investigated whether expected changes related to task engagement in P300 amplitude could be found. For this evaluation, we compared averaged activity evoked in case of a fixed ISI of 25 s (runs 1 and 2) and a fixed ISI of 15 s (runs 3 and 4) with averaged P300-related activity evoked in runs 5 and 6.

Based on the research goals, we had three hypotheses: (1) The online adaptation of the ISI reduces total runtime if compared to the long fixed ISI condition (ISI of 25 s). (2) The modulation of the ISI influences amplitudes of averaged ERP. In particular, we expect differences between ISI types with respect to peak amplitudes of the averaged ERP. (3) The usage of historic data is feasible to detect P300 in the current data (e.g., a transfer of the classifier trained on historic data to the current data is possible).

### 2.5. Analysis of subjects' behavior

#### 2.5.1. Analysis of total runtime

The total runtime was measured as the time between the first and the 30th task message within the experiment. This procedure was chosen since the total number of tasks differs slightly. This happens if the last task is from one of the categories “go to landmark” or “recharge robot” and if the adapted ISI is quite low. Solving one of these more complex tasks may take some time since the traveling distance can be rather long. Therefore, all robots may get one of these tasks. When one of the robots reaches its goal position the experiment is finished, but in this way more than 30 task messages could have been displayed to the user (see **Figure 5**).

For the statistical analysis, the value of total runtime was merged depending on the ISI type. This leads to three groups: ISI-25 (runs 1 and 2), ISI-15 (runs 3 and 4), and ISI-online adaptation (runs 5 and 6). The three ISI groups were compared by the Friedman test. For multiple comparison, the Wilcoxon signed-rank test was performed (the *p*-value was adjusted by the Bonferroni-Holm correction).

#### 2.5.2. Analysis of reaction times

To calculate the reaction times, the EEG marker files were analyzed in order to deduce all important operator- and scenario-related events. Whenever a message was presented to the operator or the operator issued a control command this was marked in the EEG file. Based on the markers we calculated the reaction times, i.e., the amount of time the operator required to react to a task message by clicking on the correct response button for the robot. Only first task messages were considered in the analysis. Repetitions of task messages were not analyzed. The median of reaction time was calculated because of strong deviations and outliers. For a comparison with the ERP average analysis an additional analysis was performed considering only reaction times after target trials with ISIs that were used for the average analysis, i.e., target trials which belonged to one of the both groups: ISI-long or ISI-short (see Table 1). Note that for the ERP analysis not all trials could be used since in run 5 6.82% of the ISI-long trials and 13.33% of the short ISI trials and in run 6 18.57% of the ISI-long trials and 12.05% of the ISI-short trials contained artifacts and were discarded from analysis.

**Table 1:** **Number of artifact-free targets for each run and distribution over different ISIs**.

**Subject**	**Number of targets for each run**					
	**Run 1**	**Run 2**	**Run 3**	**Run 4**	**Run 5**	**Run 6**
S1	26	25	28	27	32	21
S2	31	30	32	33	29	29
S3	23	19	9	27	23	17
S4	21	24	25	25	38	21
S5	23	19	25	23	25	22
S6	29	22	29	30	29	30
Average	25.50 ± 3.89	23.17 ± 4.17	24.67 ± 8.12	26.83 ± 4.02	29.33 ± 5.32	23.33 ± 5.09
**Subject**	**Number of targets for all possible ISI-groups within runs 5 and 6**					
	**Run 5**					
	**ISI-05**	**ISI-10**	**ISI-15**	**ISI-20**	**ISI-25**	**ISI-30**
S1	4	15	10	3	0	0
S2	14	7	5	2	1	0
S3	0	7	6	6	4	0
S4	0	2	10	20	6	0
S5	0	2	4	10	9	0
S6	8	12	3	4	2	0
Average	4.33 ± 5.72	7.50 ± 5.24	6.33 ± 3.01	7.50 ± 6.75	3.67 ± 3.39	0.00 ± 0.00
	**Run 6**					
S1	4	9	0	6	2	0
S2	1	12	11	3	2	0
S3	1	4	6	5	1	0
S4	0	1	2	6	7	5
S5	0	0	3	11	7	1
S6	19	6	3	0	2	0
Average	4.17 ± 7.41	5.33 ± 4.63	4.17 ± 3.87	5.17 ± 3.66	3.50 ± 2.74	1.00 ± 2.00
	**Run 5 + Run 6**					
Average	4.25 ± 6.31	6.42 ± 4.85	5.25 ± 3.49	6.33 ± 5.31	3.58 ± 2.94	0.50 ± 1.45

*For average ERP analysis different ISIs were categorized in two ISI-groups: ISI-short (marked as red) and ISI-long (marked as blue)*.

For the statistical analysis, the value of reaction time was merged depending on ISI type and this leads to three groups: ISI-25 (runs 1 and 2), ISI-15 (runs 3 and 4), and ISI-online adaptation (runs 5 and 6). The three ISI groups were compared by the Friedman test. For multiple comparison, the Wilcoxon signed-rank test was performed (the *p*-value was adjusted by the Bonferroni-Holm correction).

Additionally to median reaction times we calculated late responses after 15 s, and missed messages. EEG trials after messages with responses later than 15 s as well as missed message trials were not considered during training of the classifier (see Section 2.8).

#### 2.5.3. Questionnaires

Before the experiments started, each subject was instructed to assess its skills related to the use of computers by filling out the “Computer usage questionnaire” (CUQ) (Schroeders and Wilhelm, 2011). For the statistical analysis, the Friedman test was performed to compare the patterns of computer usages between subjects. For multiple comparison, the Wilcoxon signed-rank test was performed (the *p*-value was adjusted by the Bonferroni-Holm correction). Furthermore, *after* each of the six runs of the experimental session, the subjects had to fill out the NASA Task Load Index (TLI) questionnaire (Hart and Staveland, 1988). For the statistical analysis, the value of task load index was merged depending on the ISI type and this leads to three groups: ISI-25 (runs 1 and 2), ISI-15 (runs 3 and 4), and ISI-online adaptation (runs 5 and 6). The three ISI groups were compared by the Friedman test. For multiple comparison, the Wilcoxon signed-rank test was performed (the *p*-value was adjusted by the Bonferroni-Holm correction).

### 2.6. Analysis of the MMI behavior

The behavior of the MMI was analyzed by plotting the changes in the ISI for each subject in case of ISI adaptation (run 5 and 6, see **Figure 5**). **Figure 5** illustrates what kind of tasks were presented to the operator and which ISI was used, therefore the trace is the same as it was during the actual experiment. The purpose of this analysis was to give an impression of how “good” the adaptation worked and which ISI was most comfortable for the operator over the course of the run. For a comparison of the mean ISI between subjects, the mean ISI for each subject and run was calculated and the mean ISI of each run was compared between subjects by using the Friedman test. For a multiple comparison, the Wilcoxon signed-rank test was performed (the *p*-value was adjusted by the Bonferroni-Holm correction). Furthermore, we investigated whether the mean ISI is a useful indicator for the analysis of the MMI behaviors. To this end, the correlation between the mean ISI and the total runtime was calculated using the Spearman's rank correlation. We expected a positive correlation such that a longer ISI leads to a longer total runtime. In addition, we investigated *task type* as another factor with a potential effect on the total runtime. For example, the task types “go to landmark” and “charging robot” required a longer total runtime compared to the task type “send message.” The frequency and order of task types were randomly chosen. Thus, differences in frequency of task types can in principle lead to differences in total runtime between subjects. However, we did not expect a strong correlation between task type and total runtime.

### 2.7. ERP-average analysis

Continuous EEGs were bandpass-filtered (0.1–30 Hz) and segmented into “target” trials from −100 to 1000 ms with respect to the stimulus onset (baseline correction: from −100 ms before the stimulus onset to 0 ms). As for the machine learning analysis only trials after the first task messages which have been responded to within a time period of 15 s were labeled as “target” trials when analyzing runs 1–4. For runs 5 and 6 again only trials with answered task messages were used as “target” trials and averaged as explained in Table 1. This procedure copies the procedure of the offline analysis. Trials after missed task messages were not averaged to exclude their contribution to the average ERP characteristic. We used a common average reference (CAR) and recalculated the data from channel FCz. For ERP average analysis only artifact-free segments were used (see Table 1). Artifact detection was performed semi-autonomously with a maximum amplitude of −100 μV and 100 μV. We compared average artifact-free ERP activity evoked in runs with ISI-25 and ISI-15 as well as ISI-long and ISI-short. Trials for ISI-25 were conducted in runs 1 and 2 and trials for ISI-15 in runs 3 and 4. An adaptation of the ISI in runs 5 and 6 did not only result in various ISIs but also in individual ranges of ISIs for different users (see Table 1). Therefore, we individually divided the EEG segments of runs 5 and 6 into two ISI groups with respect to trials being evoked after short or long ISIs for each subject. For example, from the data of the subject depicted in **Figure 9** we merged examples after ISI-15 and ISI-20 to calculate average ERP activity after long ISIs and ISI-5 and ISI-10 to calculate average ERP activity after short ISIs (see Table 1). By means of this procedure, we could compare averaged P300-related activity for ISI-short and ISI-long of runs 5 and 6 with the activity evoked in runs 1 and 2 (fixed ISI of 25 ms: ISI-25) or runs 3 and 4 (fixed ISI of 15 ms: ISI-15) (Table 2.2). For peak detection, we selected a single window of the interval 0.3 –0.7 s after a “target” trial. The positive maximum peak was detected within the selected window.

Table 2**Design for the recording of EEG data, evaluation design for ERP analysis and design for the analysis of single-trial classification performance (online/offline-mode)**.

**Table:** 

**Table 2.1. EEG data**	**Table 2.2. Evaluation design for ERP analysis**	
(a) run 1: fixed ISI of 25 s	Average ERP in (a)	ISI-25: average of (a) and (b)
(b) run 2: fixed ISI of 25 s	Average ERP in (b)	
(c) run 3: fixed ISI of 15 s	Average ERP in (c)	ISI-15: average of (c) and (d)
(d) run 4: fixed ISI of 15 s	Average ERP in (d)	
(e) run 5: online adapted ISI	Average ERP in (e)	Various ISIs are grouped in short and long ISI
(f) run 6: online adapted ISI	Average ERP in (f)	for each subject: (e), (f), or average of (e) and (f)

**Table:** 

**Table 2.3. Online stCL**		**Table 2.4. Offline stCL**							
**Adapted ISI classifier transfer**		**Adapted ISI (e) transfer**		**ISI-25 (control) no transfer**		**Adapted ISI (f) transfer**		**ISI-15 (control) redno transfer**	
**Training**	**Test**	**Training**	**Test**	**Training**	**Test**	**Training**	**Test**	**Training**	**Test**
ISI-25 (fixed ISI of 25 s)	(e)	(a)	(e)			(c)	(e)		
(a) + (b) merged		(b)	(e)			(d)	(e)		
			Mean (e)	(a)	(b)		Mean (e)	(c)	(d)
ISI-15 (fixed ISI of 15 s)	(f)	(a)	(f)			(c)	(f)		
(c) + (d) merged		(b)	(f)			(d)	(f)		
			Mean (f)	(b)	(a)		Mean (f)	(d)	(c)

*ERP, event-related potentials; online stCL, online single-trial classification; offline stCL, offline single-trial classification; and ISI, inter-stimulus interval. Each run contained 30 trials. For online single-trial classification, 60 trials (e.g., runs 1 and 2) were used to train a classifier and 30 trials (e.g., run 5) were used for evaluation. For offline single-trial classification, 30 trials were used for training and testing in both cases (no transfer/classifier transfer)*.

For the statistical analysis of average ERP amplitude values with a sample size of 6 (i.e., 6 subjects), we performed the Wilcoxon signed-rank test to compare different ISI types (ISI-25 vs. ISI-15 and ISI-long vs. ISI-short).

### 2.8. Machine learning analysis

**Figure 4:**
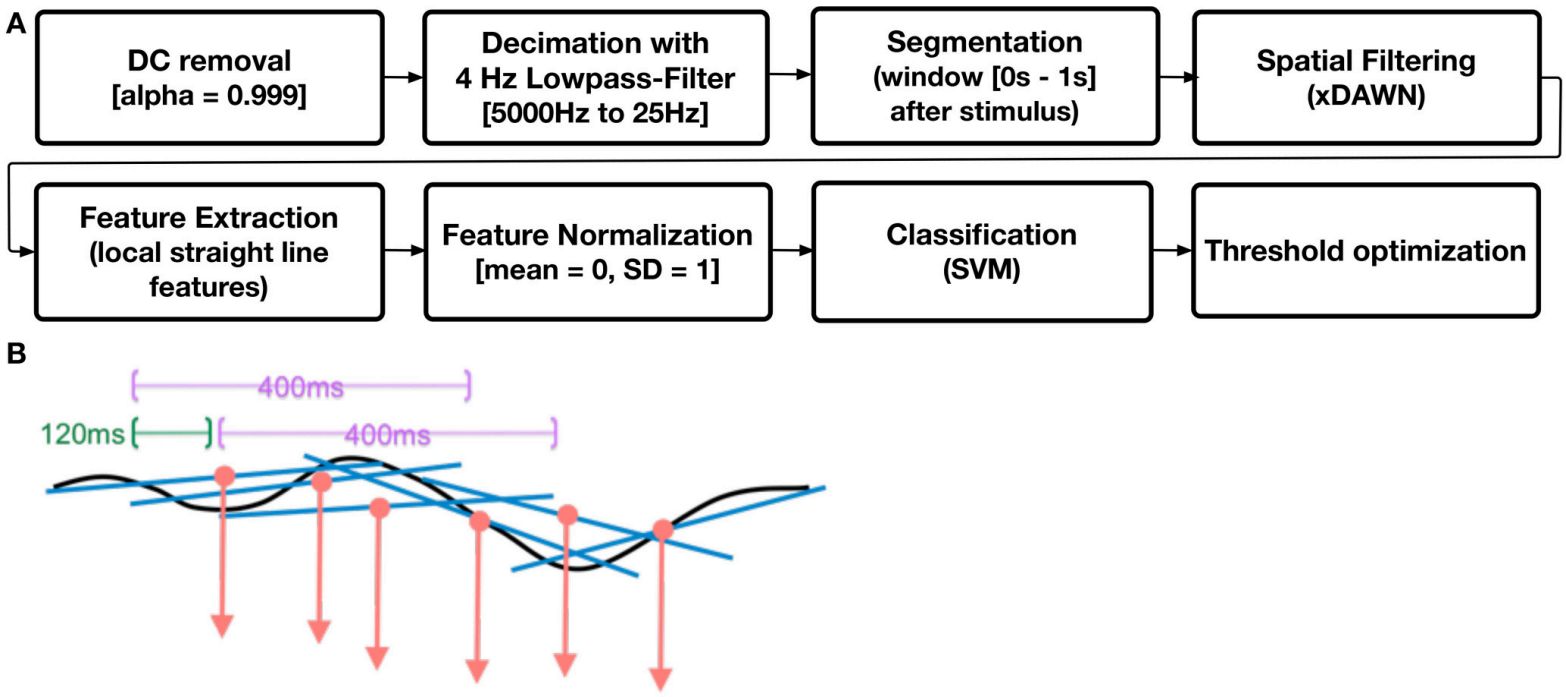
**Data Processing. (A)** data flow for signal processing and single-trial classification. **(B)** example of an ERP (black line) being processed as local slopes of a straight line.

The data flow of the machine learning algorithm is depicted in Figure 4A. For the analysis the software framework pySPACE (Krell et al., 2013a) was used. First the continuous EEGs were processed by a DC removal filter, which is an online-capable method for centering the signal around zero. The normalized EEGs then were decimated from 5000 to 25 Hz.A cutoff frequency of 4 Hz was used for the anti-alias filter in the decimation process (Jansen et al., 2004; Ghaderi et al., 2014). Afterwards the EEGs were segmented into chunks of 1 s length. Chunks cut right after a *first* task message (*not* after repetitions of messages) were labeled as “*targets.”* Within the training, these windows were only cut if the operator *responded* to the first task message within 15 s after presentation, in the online case *every* first task message was analyzed. We further cut “*standard”* windows of length 1 s while training. These windows were needed to train the used binary classifier. The standard windows were cut every second with the constraint that no other action relevant for task recognition was performed in a range from [−1, 1] s around the cut window. For the task recognition, actions such as the presentation of a task message or the response of the operator of one of these messages were used. The segments were further processed with the xDAWN spatial filter (Rivet et al., 2009). The xDAWN is a spatial filter especially designed for P300 detection. It (1) enhances the separability of the P300 ERP and noise and (2) reduces the dimensionality of the data. To achieve this, a set of filters maximizing the signal-to-signal-plus-noise ratio is computed on a training data set. The resulting filters can be used to create a set of pseudo-channels that contain the filtered signal. From the newly created pseudo channels the 8 most relevant channels were used for further processing.

As features we used local straight line features, i.e., polynomial features. To fit a polynominal function EEG data must be segmented (see Figure 4B). Earlier investigations showed that the longer the segments are chosen, the more coefficients are needed to keep the performance level high. For this paper every 120 ms, segments of length of 400 ms within the 1 s segments after stimulus onset were cut. Polynominal features of order one, i.e., straight lines were fitted to the 400 ms long segments of the ERP data with 120 ms steps to describe the ERP in terms of a series of slope values (see Figure 4B). Polynominal features of order one have been chosen since in former investigations of P300 ERP activity the highest value was obtained with this low coefficient. Previous analyses, too, as performed for example in Wöhrle and Kirchner (2014b) support our choice.

After this preprocessing a Support Vector Machine (SVM) (Chang and Lin, 2011) was used as classifier. During training the complexity of the SVM was optimized with a grid search and an internal five-fold cross validation. The possible complexities were 10^*n*^ with *n* ∈ 0, −1, …, −6. Further a threshold optimization was applied (Metzen and Kirchner, 2011). Further a threshold optimization was applied (Metzen and Kirchner, 2011). After building the model of a SVM the decision boundary is defined as 0 and the two classes (here target and standard) are at the positive and negative side of the boundary. The threshold optimizations gives the opportunity to further improve the classification performance with respect to a given metric, here the balanced accuracy. The threshold is shifted into the negative or positive direction, in a way that for the training data the highest classification performance in terms of balanced accuracy is achieved.

We used the balanced accuracy (bACC), i.e., the mean of true positive rate (TPR) and true negative rate (TNR), as the performance metric due to the insensitivity of this metric to changes in class distribution (Krell et al., 2013b; Straube and Krell, 2014). Area under the curve (AUC) values were additionally calculated. Classification performance was compared between all conditions. For details see Table 2.3. Although the adaptation of the ISI was evaluated online (Table 2.3: online stCL), we additionally analyzed the data in the offline mode (Table 2.4: offline stCL). This procedure was chosen for reasons of fair comparison. While in the online mode data of two runs (runs 1 and 2 or runs 3 and 4) were used for training, this was not possible for evaluating the general P300 detectability in case of fixed ISIs since here only one run could be used for training while the other was used for testing. By means of the chosen offline approach we were able to analyze the no-transfer case (as baseline/control) and the transfer case equally.

For the statistical analysis on single-trial classification performance, two separate comparisons were performed by using the Wilcoxon signed-rank test. First, we compared two online cases: online P300 detection in run 5 vs. run 6 (see (e) vs. (f) in Table 2.3: online stCL). Here, two samples per subject were obtained for each online case. Altogether, we obtained a sample size of 12 (2 samples × 6 subjects) for each online case. Second, two adapted ISI conditions were compared with two fixed ISI-conditions in offline mode depending on the type of training data (ISI-25 or ISI-15) used to train the classifier: (1) adapted ISI (e) vs. ISI-25 (control) (see in Table 2.4: offline stCL) and (2) adapted ISI (f) vs. ISI-15 (control) (see in Table 2.4: offline stCL). In the offline analysis, the number of training examples for the fixed ISI conditions (run 1 or run 2 / run 3 or run 4, see Table 2.4) was half the number of training examples used for the adapted ISI conditions in case of online evaluation (run 5 or run 6, see Table 2.3). For a fair comparison between the adapted and fixed ISI-condition, only one run (run 1 or run 2) was used to train the classifier to test it on run 5, and the mean of classification performance obtained by using run 1 or run 2 for training was calculated in the case of the *adapted ISI(e)* (see Table 2.4 (e) in offline stCL). Similarly, in the case of the *adapted ISI(f)*, only one run (run 3 or run 4) was used to train the classifier to test it on run 6 and the mean of classification performance obtained by using run 3 or run 4 for training was calculated (see Table 2.4 (f) in offline stCL). Each adapted and fixed condition has two samples per subject. Altogether, we obtained a sample size of 12 (2 samples × 6 subjects) for each condition.

## 3. Results

### 3.1. Behavior of subjects

#### 3.1.1. Total runtime

**Figure 5:**
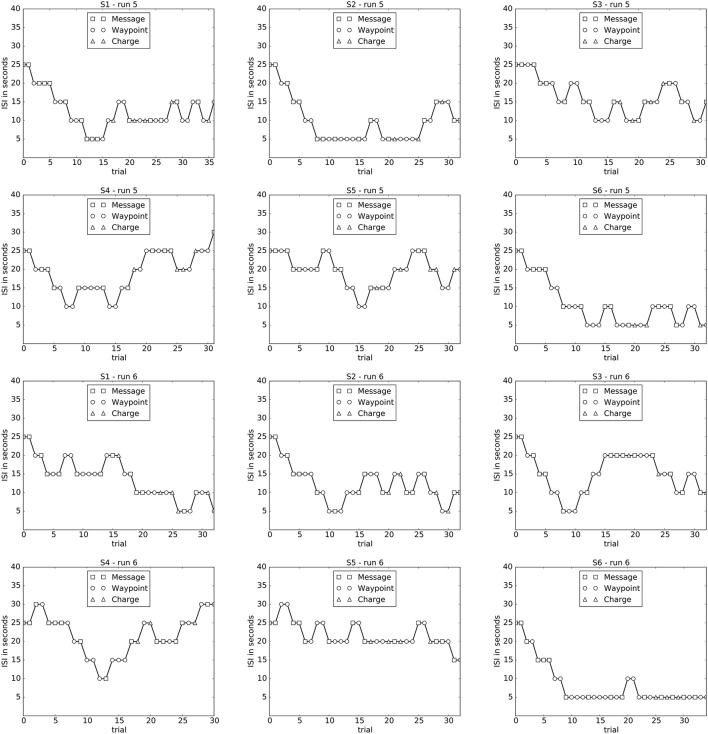
**Changes in ISI over each run in case of the adapted ISI condition (runs 5 and 6) for each subject are depicted**.

**Figure 6:**
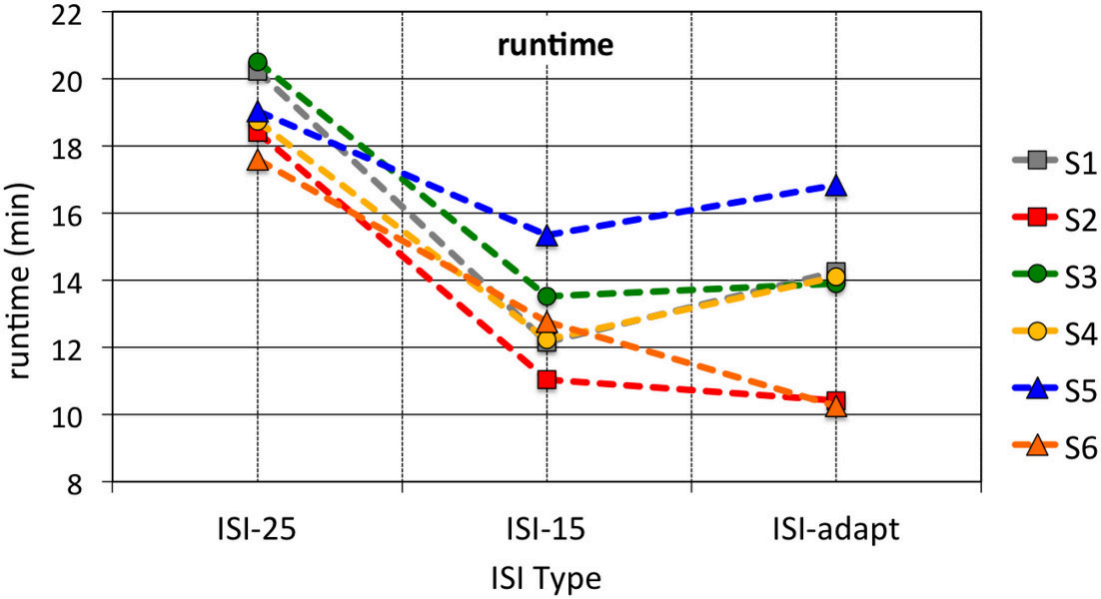
**Mean runtime for different ISI conditions**. The means of both runs for each ISI type are depicted. The median across all subjects for each ISI type was 17.59 for ISI-25, 11.49 for ISI-15 and 12.45 for ISI-adapt.

Figure 5 shows how the ISI changed over one run based on the inferred task load and task engagement of the user measured by P300 detectability. Subjects reported that the online adaptation made them feel to have just the right task frequency. This indicates that online adaptation of the MMI has a positive effect on the interaction. The finding was supported by the results of the behavioral analysis of the total runtime (see Figure 6). The online adaptation of the ISI reduced total runtime significantly if compared to the ISI-25 condition [*p* < 0.001]. Moreover, there was no significant difference in total runtime between the case of online adaptation of ISI and the case of ISI-15 condition [*p* = *n*.*s*.].

#### 3.1.2. Reaction time

**Figure 7:**
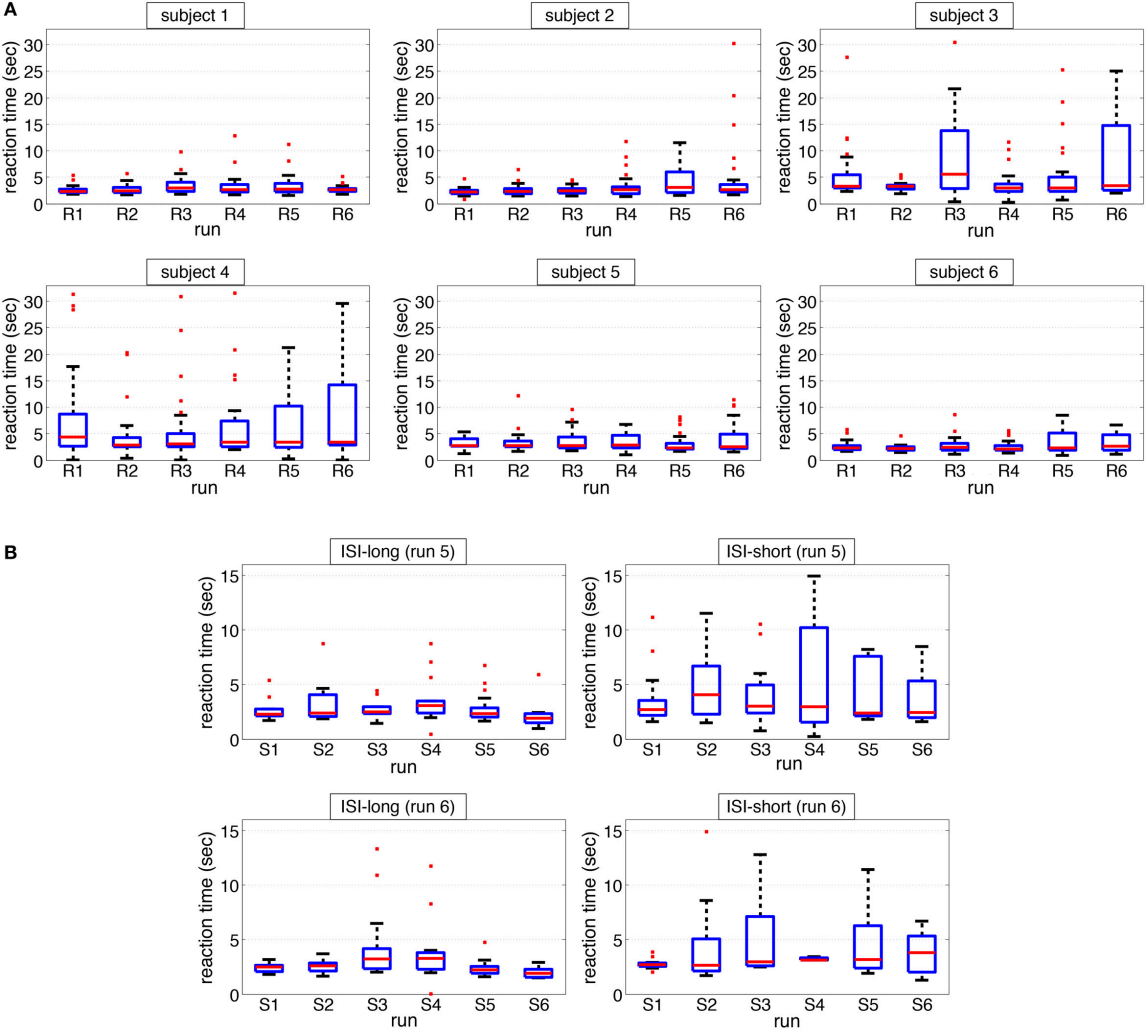
**Median response time. (A)** median reaction times for each run and each subject are depicted. **(B)** median reaction times for each run and each subject sorted with respect to trials with short and long ISI as defined for average ERP analysis are depicted.

Figure 7A shows the median reaction time for individual subjects over all runs. It can be seen that median reaction times are very similar over all conditions and runs for each subject. When merging the two runs of each condition (ISI-25, ISI-15, and ISI-adapt) we found no significant difference between ISI types. However, when analyzing median reaction time individually for ISI-long and ISI-short groups of runs 5 and 6 as performed for average ERP analysis it can be seen that the reaction time on task messages presented after short ISIs showed a higher variance compared to task messages presented after long ISIs (see Figure 7B).

**Figure 8:**
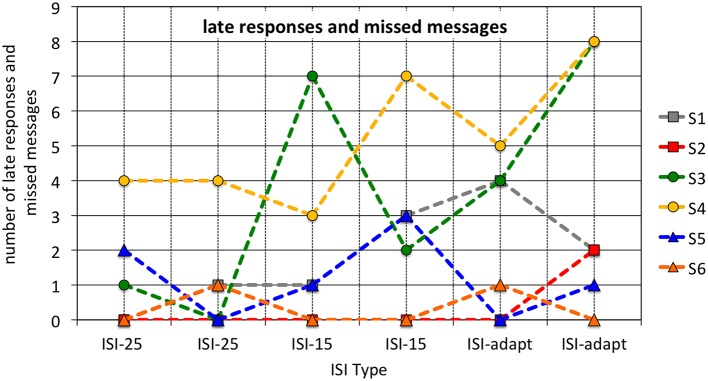
**Sum of late responses and missed messages for each run and each subject is depicted**.

A descriptive analysis of the sum of late responses and missed messages per subject for each run is visualized in Figure 8. It can be seen that for some subjects the number of late responses and missed messages was higher than for others (subjects 3 and 4). Table 3 provides information about the number of late responses, missed messages and the sum of both as depicted in Figure 8.

**Table 3:** **Number of tasks with late or no response in runs 5 and 6**.

	**Run 5**			**Run 6**		
**Subject**	**Late**	**Missed**	**Total**	**Late**	**Missed**	**Total**
S1	4	0	4	2	0	2
S2	0	0	0	2	2	4
S3	4	0	4	8	0	4
S4	5	0	5	8	0	8
S5	0	1	1	1	0	1
S6	1	0	1	0	2	2

#### 3.1.3. Questionnaires

The analysis of the “computer usage questionnaire” shows a significant difference between subjects, especially subject 4 differed significantly from the other subjects [*p* < 0.03]. The analysis of the “NASA Task Load Index (TLI) questionnaire” shows no significant differences between runs [*p* = *n*.*s*].

### 3.2. Behavior of MMI

Figure 5 depicts the changes of the ISI for both adapted runs (runs 5 and 6) for each subject. It can be seen that the adaptation of the ISI is very individual for each subject and even for each run. While for some subjects and runs, as for subject 2 in run 5, the ISI goes down to the minimum of 5 s and stays there for almost 20 trials, for other subjects the ISI is not reduced that much (see for example subject 5 for both runs).

In most cases the ISI gradually decreases just to later increase. However, there are exceptions from these findings. For example subject 1 shows a reduction of ISI at the end of run 6 and subject 6 stays with a low ISI during both runs. For all subjects the ISI starting with 25 s was reduced to a lower mean ISI with average values of 14.67 and 15.62 s (runs 5 and 6) (see **Table 5**). Moreover, we could also find differences in the mean ISI between subjects. For example, while the mean ISI for subject 4 and subject 5 is around 19 and 22 s (runs 5 and 6), the mean ISI for subject 6 is at 10.45 and 8.43 s (runs 5 and 6) and for subject 2 at 9.85 and 12.42 s (runs 5 and 6). The mean ISI for Subject 4 and subject 5 was significantly higher compared to the other subjects [*p* < 0.017]. Furthermore, the mean ISI correlated strongly with the total runtime [*r* = 0.874, *p* < 0001], but not the task type (e.g., send message, go landmark, etc.).

### 3.3. Average P300-related activity

**Figure 9:**
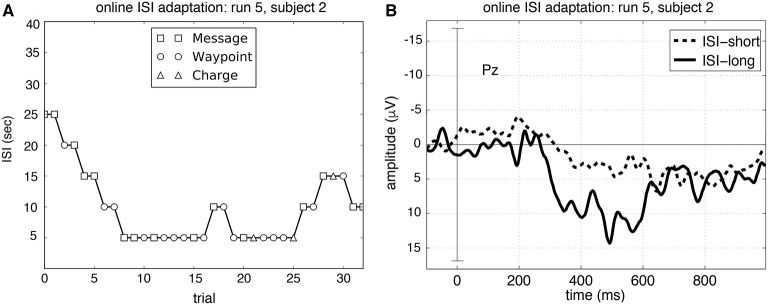
**Adaptation of the ISI over one run and the evoked averaged ERP activity at Pz for one subject: (A) depicts online adaptation of ISI in case of using the classifier trained on data with ISI of 25 s (i.e., training data: ISI-25, test data: run 5, see (e) in Table 2.3: online stCL) and (B) the corresponding averaged ERP curve evoked during the same run (Table 2.2: ERP analysis)**. Only artifact-free trials were used: 7 trials for ISI of 15 s and ISI of 20 s; 21 trials for ISI of 5 s and ISI of 10 s. Different types of tasks (tasks of type: message, way point and charging, see Figure 3 for details) had to be solved by the subjects.

**Figure 10:**
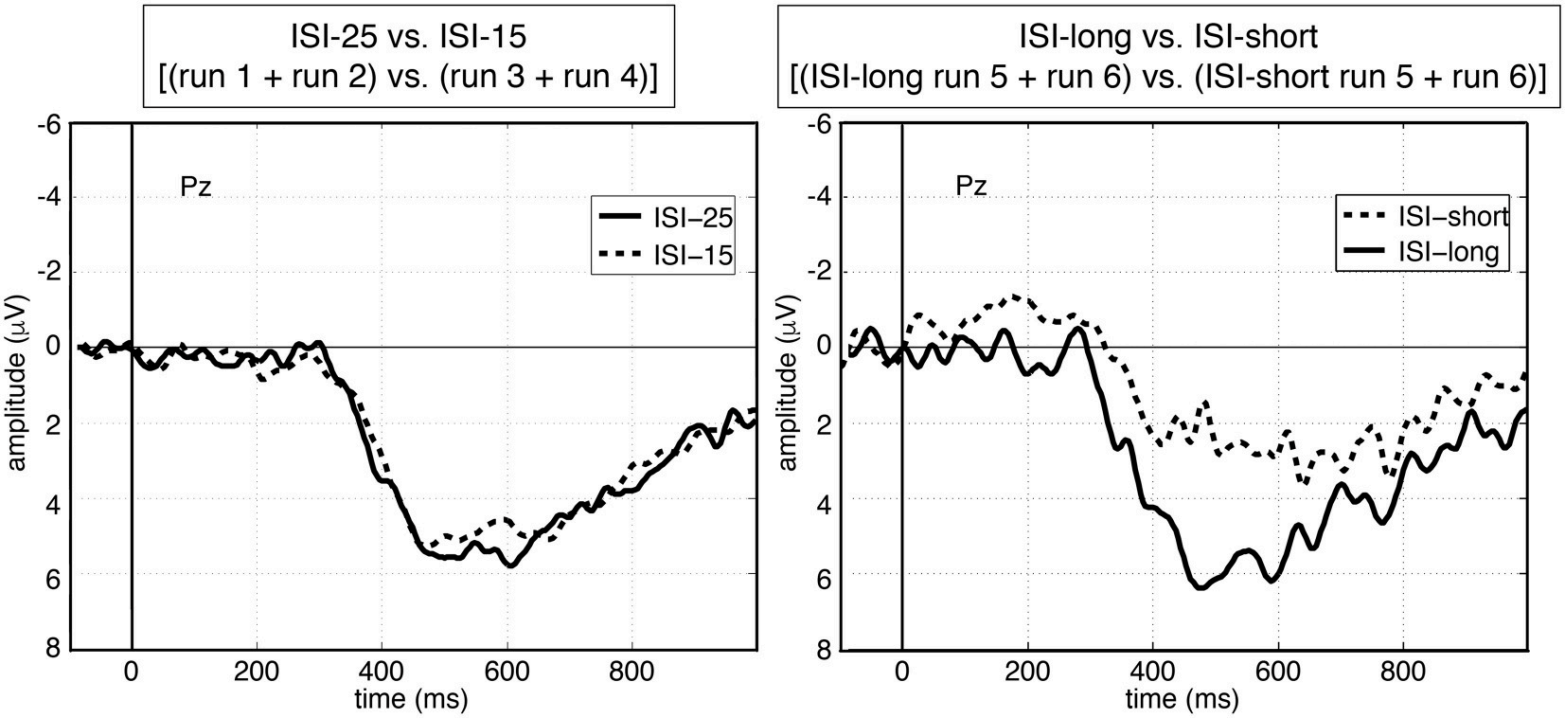
**Averaged ERP activity over all subjects at electrode Pz under offline condition (left side) and under online condition (right side)**. Grand averages over all subjects are depicted. Each run contained 30 trials. Only artifact-free trials were used: 292 trials for ISI-25 and 313 trials for ISI-15, 139 trials for ISI-long and 164 trials for ISI-short.

As shown in Figures 9,10, we observed differences in averaged ERP shape depending on the ISI condition (short/long ISI). Note that the ISI in case of long ISIs and short ISIs differ for both average analysis conditions (fixed-ISI condition and adapted-ISI condition, see **Table 5**). While for ISI-long average analysis condition the ISI is set to 25 s, ISI-long for the adapted-ISI condition is around 19 s. Similar differences can be found for the ISI-short average analysis condition (fixed short ISI: 15 s versus adapted ISI around 10 s). The peak amplitude of the averaged P300-related activity was not significantly reduced in case of ISI-15 (runs 3 and 4) compared to ISI-25 condition (runs 1 and 2) [*p* = *n*.*s*.]. However, we observed a significant reduction in averaged P300 amplitude in run 5 and run 6 for short ISI groups compared to long ISI groups [*p* < 0.04]. Furthermore, there was a significant difference between ISI-15 and ISI-short [*p* < 0.04], but not between ISI-25 and ISI-long [*p* = *n*.*s*.].

### 3.4. Online P300 detectability

Finally, we achieved high classification performances in both the online and offline analysis. In the online evaluation, we found no significant difference between both online runs [adapted ISI (e) vs. adapted ISI (f): bACC of 0.77 vs. bACC of 0.78, *p* = *n*.*s*., see adapted ISI (e) vs. adapted ISI (f) in Table 4.1]. In the offline evaluation, classification performance obtained by using the classifier trained on ISI-25 statistically differed from classification performance obtained in case of no transfer [ISI-25 vs. adapted ISI: bACC of 0.84 vs. bACC of 0.75: *p* < 0.003, see adapted ISI (e) vs. ISI-25 in Table 4.2]. However, we found no significant difference in classification performance when using the classifier trained on ISI-15 compared to the case of no transfer (ISI-15) [ISI-15 vs. adapted ISI: bACC of 0.80 vs. bACC of 0.79: *p* = *n*.*s*., see adapted ISI (f) vs. ISI-15 in Table 4.2]. There was no significant difference between the online and offline evaluation for the case of ISI-adaptation [adapted ISI (e) in Table 4.1 vs. adapted ISI (e) in Table 4.2: *p* = *n.s*. ; adapted ISI (f) in Table 4.1 vs. adapted ISI (f) in Table 4.2: *p* = *n*.*s*.]. In summary, we found a transfer effect on classification performance in case that the classifier was trained on data from the ISI-25 runs. However, such an effect was missing when the classifier was trained on data from the ISI-15 runs. It must be emphasized that the classification performance was very similar in case of both classifier transfer analyses, i.e., adapted ISI (e) and adapted ISI (f) (see Table 4.1).

Table 4**Online and offline classification performance**.

**Table:** 

**Table 4.1. Online single trial classification performances (cf. Table 2-3. Online stCL)**								
	**bACC**				**AUC**			
	**Adapted ISI (e) classifier transfer**		**Adapted ISI (f) classifier transfer**		**Adapted ISI (e) classifier transfer**		**Adapted ISI (f) classifier transfer**	
S1	0.7646		0.7586		0.8481		0.8343	
S2	0.8403		0.8177		0.8692		0.8596	
S3	0.7892		0.7422		0.8516		0.8681	
S4	0.6486		0.7083		0.7620		0.7365	
S5	0.7981		0.7631		0.8790		0.8621	
S6	0.7931		0.9021		0.9292		0.9375	
Mean	0.7723		0.7820		0.8565		0.8497

**Table:** 

**Table 4.2. Offline single trial classification performances (cf. Table 2.4. Offline stCL)**								
	**bACC**				**AUC**			
	**Adapted ISI (e) transfer**	**ISI-25 no transfer**	**Adapted ISI (f) transfer**	**ISI-15 no transfer**	**Adapted ISI (e) transfer**	**ISI-25 no transfer**	**Adapted ISI (f) transfer**	**ISI-15 no transfer**
S1	0.7057	0.8086	0.7595	0.7725	0.8063	0.8815	0.7979	0.7966
S2	0.7690	0.9536	0.8366	0.9361	0.8183	0.9873	0.8870	0.9604
S3	0.6987	0.7568	0.7912	0.7406	0.8643	0.8923	0.476	0.8159
S4	0.6772	0.7310	0.7195	0.7187	0.7670	0.7451	0.7170	0.8459
S5	0.7722	0.8135	0.7685	0.7650	0.8054	0.8942	0.8193	0.8745
S6	0.8625	0.9600	0.8843	0.8864	0.9037	0.9692	0.9549	0.9045
Mean	0.7476	0.8373	0.7933	0.8032	0.8275	0.8951	0.8373	0.8663

## 4. Discussion

### 4.1. Improvement of interaction

Supporting our hypothesis (1) behavioral data showed that total runtime in runs with adapted ISI was significantly shorter compared to an unadapted condition with an ISI of 25 s. Although there was no significant difference between the adapted ISI and the fixed shorter ISI of 15 s the mean total runtime was still very low considering the fact that runs with ISI adaptation did start at an ISI of 25 s. Significant differences in the total runtime between runs with adapted ISI and the fixed shorter ISI of 15 s were not expected, since the time needed until a task was performed by a robot does (although not strongly) depend on the type of task. For example, sending data was very fast and instant while reaching a certain landmark could take a long time depending on the current position of the robot and the landmark. Thus, some deviation in runtime depending on the kind of tasks that had to be performed by the robot, was expected. On the other hand, we did not choose subjects with a certain qualification but chose subjects independent of their experience in robot control or video gaming. Thus, we expected differences in the subjects' performances resulting in different “suitable” ISIs and hence also in different total runtimes. Important was that a significantly shorter runtime could be achieved compared to the fixed ISI-25 condition under which all the subjects could perform the tasks without being stressed.

Besides, the goal was not to reduce the total runtime to a minimum but to adapt the ISI with respect to the demands of the user of the MMI. Indeed, for some subjects the mean ISI was reduced to mean values around 10 s while for other subjects, i.e., subjects 4 and 5, the ISI was clearly above 15 s (around 19 s, see Section 3.2). On the other hand, even for subjects for whom the ISI was not reduced that much, mean ISI was clearly below 25 s, supporting our presupposition from the 4 test subjects that were not included in this study that a fixed ISI of 25 s ensures that all subject can easily perform the tasks but will probably make the subjects feel bored. An interesting finding is that subject 4 for which the ISI was reduced only to a still high value (around 19 s) significantly differed from the other subjects with respect to computer usage as evaluated by the “Computer usage questionnaire” (CUQ). This finding supports our assumption that the MMI could be adapted based on the detectability of the P300 to support the user with respect to her or his general capabilities. Note that subject 4 showed the lowest classification performance in both runs compared to the other subjects (although no significant differences between subjects could be found, see Table 4). Moreover, subject 4 had a high amount of late responses and missed messages (see Figure 8). Another interesting finding is that the median reaction time does not significantly differ between subjects. This finding suggests that in our application behavioral data is probably not a good indicator for task load. Moreover, it shows that using our approach subjects were exposed to an appropriate workload. In summary, the results suggest that by using the developed MMI utilizing embedded Brain Reading, the MMI cannot only be adapted to the general capabilities of the user (e.g., experienced or rather inexperienced in computer usage) but also to the changes in task load over time.

### 4.2. Changes in the characteristic of average P300 depending on the ISI

Applying average ERP analysis, we were able to show that during a complex multi-robot control task a P300-related activity is evoked by task messages which are presented to the operator. This finding is the most important basis for our approach to adapt an MMI based on P300 detectability. As expected we found *no* significant differences in the averaged-peak P300 amplitude for both fixed ISI conditions. This supports earlier findings that the ISI has no influence on the P300 amplitude in case of long ISIs (longer than 6–8 s as found by Polich, 2007). More importantly this finding supports our assumption that on both fixed ISI conditions the general workload on the subjects was rather modest and comparable. Hence, any found differences in the P300 peak amplitude should be caused by changes in the current task load and task engagement. This finding is supported by the fact that in case of an ISI adaptation the average P300 peak amplitude was significantly reduced for trials after short ISIs compared to trials after long ISIs.

Our results from the average ERP analysis support hypothesis (2): we could show differences in the P300 peak amplitude for average conditions with a high task load (averaged ERP activity after ISI-long in adapted ISI condition) compared to average conditions with low task load (averaged ERP activity after ISI-long in adapted ISI condition).

The finding that the peak amplitude of the average P300 activity after trials with ISI-short (adapted ISI condition) is significantly smaller compared to the peak amplitude of the average P300 activity of both fixed ISI conditions (ISI-25 and ISI-15) suggests that for all subjects the MMI was indeed adapted to achieve the best performance without enhancing the workload too much such that no P300 would be evoked. Tests on 4 subjects (not included in this study) showed that in cases in which the workload was too high no P300 was evoked on average or could not be detected in single-trial while subjects reported that they were very stressed and could not perform the tasks. Hence, the MMI is adapted such that subjects perform best while avoiding an excessive general workload. Some subjects were able to keep their performance high with a short ISI all through the experiment while others did not. For the latter, the MMI was again adapted to longer ISIs reducing the task load back to normal. The task load and thus the general workload being modest under the adapted condition after long ISIs is supported by the finding that the average P300 peak amplitude evoked after long ISI trials under the adapted ISI condition is comparable to the average P300 peak amplitude under the fixed ISI conditions (ISI-25 and ISI-15). This was even the case although the mean long and short ISI differed strongly between subjects (see Tables 1, 5). Based on these findings we suggest that the P300 ERP is indeed a good indicator for the current and individually different task load of a subject while controlling the robots.

**Table 5:** **Mean ISIs in case of online ISI-adaptation (runs 5 and 6)**.

	**Mean ISI in sec**.					
	**Table 5.1**		**Table 5.2**			
**Subject**	**Run 5**	**Run 6**	**Run 5**		**Run 6**	
			**ISI-long**	**ISI-short**	**ISI-long**	**ISI-short**
S1	12.7 ± 5.01	13.94 ± 5.47	16.15	8.95	21.25	8.46
S2	9.85 ± 5.97	12.42 ± 5.09	16.47	6.67	16.07	9.62
S3	16.25 ± 4.84	15.15 ± 5.43	22.00	12.31	17.27	9.00
S4	19.22 ± 5.46	21.94 ± 5.63	21.15	14.17	22.69	13.33
S5	19.55 ± 4.33	21.82 ± 3.44	22.37	13.33	25.63	8.57
S6	10.45 ± 6.08	8.43 ± 5.95	17.86	8.00	15.00	6.20
Average	14.67 ± 4.29	15.62 ± 5.35	19.33 ± 2.84	10.57 ± 3.10	19.65 ± 4.19	9.20 ± 2.33

*Table 5.1: mean over all trials. Table 5.2: mean over a selected group of trials with ISI-short and ISI-long as defined for average ERP analysis (see Table 1)*.

### 4.3. Detectability of P300 in single-trial

The results of the offline machine learning analysis support that the P300-related activity which was evoked by task messages can be detected in single-trial even in case that the classifier is transferred between different ISI conditions. Thus, the results support hypothesis (3).

When comparing online classification with offline classification a performance drop can be observed. This can be explained as follows: In the online case each first message was classified independently of having been responded to. Therefore, trials after missed task messages which likely did not contain a P300 were classified, leading to “false negative” results. It was therefore expected that classification performance was lower for the online case, since the approach is sensitive to missed targets. The small difference between online and offline results support that the MMI was well designed such that only few target events (messages) were completely missed (see also Table 3).

Besides this, in both transfer cases similar classification performance can be achieved. Hence, for an application it is not that relevant for the classification on which data a classifier is trained. While we found no significant differences between subjects for online classification performance it is noticeable that subject 4 had the worst classification performance in both runs compared to the other subjects (Discussion see Section 4.1).

### 4.4. P300 detectability as index for task load or task engagement

By reducing the ISI to way shorter ISIs compared to the ISI-15 condition (see Table 5) we strongly enhanced the task load and likelihood of conflicts since subjects might still be engaged in a former task when a new task message was presented. This is supported by two findings: (1) the higher variance in reaction time found for the ISI-short group (based on grouping for average analysis) and (2) the smaller average P300 evoked after short ISI trials in the adapted ISI condition (see Figure 10). Likely, subjects were still involved in a previous task and often could therefore respond to a new task only with a delay.

We found a similar effect in a previous study (Kim and Kirchner, 2012). In this previous study, subjects played a labyrinth game and had to respond to target stimuli which were presented in an oddball design. However, subjects were not allowed to respond to target events right away. We asked the subjects to steer the ball in a save corner first before answering a target event. When analyzing the average P300 potential we grouped the data with respect to reaction time such that the first group consisted of EEG trials with only short reaction times up to 1.4 s, for the second group trials were added which had reaction times up to 1.6 s, for the third group up to 1.8 s, the fourth up to 2.0 s, and the fifth up to 7.0 s. Although keeping the trials with short reaction times up to 1.4 s for the second group and up to 1.6 s for the third group, we still found descriptive differences in average peak amplitude of the P300 component between all groups with highest amplitude for the group of 1.4 s and lowest for the group of 7.0 s. When classifying between standard and target trials we found significant differences between the group of 1.4 s compared to all other groups with the exception of group 1.4 s compared to group 1.6 s and significant differences between the group of 7.0 s compared to all other groups with highest classification performance of 0.85 for the group of 1.4 s and lowest classification performance of 0.76 for the group of 7.0 s. These results suggest that ongoing task engagement, i.e., playing the labyrinth game, reduced the P300 evoked by a new target stimulus tremendously and would also reduce classification performance.

### 4.5. Summary and outlook

In summary, our results show that complex interaction between humans and robotic systems can be improved by the application of an MMI adapted by eBR. The time between tasks can be adjusted such that a reduction of run time compared to a safe mode is possible. The strength of adaptation does further correlate with the experience of the user. Thus, the MMI can be adapted to the needs of the user within a range of workload that can otherwise not be resolved. Our approach shows that EEG activity like the P300-related activity that is naturally evoked during interaction can be used to adapt an MMI with respect to online changes in task load or task engagement of an operator. Thus, the dual-task design (with a primary and usually artificially introduced secondary task) that is often applied to infer on current processing capacity of the brain must not be applied to adapt for task engagement. The ERP activity can be used rather naturally, similar to approaches that make use of ratios of EEG power bands (Pope et al., 1995) while being specific to certain stages of information processing (Prinzel et al., 2003). Hence, for the user, our approach of measuring brain states and task engagement remains invisible and avoids any possible additional load on the user, since the task itself is used to measure task load, without any additional task.

In the future, we will have a closer look at the long term effect of adaptation of the ISI compared to a high task load condition, i.e., ISI of 10 s or even lower. For this, it is required to avoid the recording of extra training data since this requires a considerable amount of time. The total time for one experiment (6 runs) was already between three to 4 h including preparation. Thus, for a long term study, preparation and especially training of the classifier must be kept to a minimum. This can be achieved by using zero-training approaches (Krauledat et al., 2008; Kindermans et al., 2012) or by using old training data from either previous recordings of the same subject or other subjects (Lotte and Guan, 2010; Devlaminck et al., 2011; Samek et al., 2013). To reduce transfer effects (between sessions and between subjects) adaptive algorithms for the spatial filter (Rivet et al., 2011; Ghaderi and Straube, 2013), the classifier (Li et al., 2008; Lu et al., 2009; Tabie et al., 2014) or both (Wöhrle et al., 2015) can be applied. Moreover, we want to investigate whether adaptive measures can be used to even improve the classification performance and the support for the user as we could already show for the prediction of movement onsets (Tabie et al., 2014). Finally, we will investigate transferability of the final approach to a mobile analysis system which makes use of hardware accelerators as already tested for the current application. Even for an adaptive approach hardware accelerators have shown to be feasible for the detection of both the P300 event-related potential (Wöhrle et al., 2013b,a, 2014a) and the movement-related ERP activity (Wöhrle et al., 2014b).

## Author contributions

EK developed concepts for the MMI and for data evaluation, interpreted the results and wrote most of the manuscript. She further contributed to data recording, evaluation and statistical design. SK performed the analysis of ERP averages and of questionnaires and the statistic evaluation of classification performance, ERP results and behavior data. She further wrote parts of the manuscript and supported data acquisition. MT conducted the experiments, designed the online processing flow, did the machine learning analysis and evaluated the ISI changes. He further wrote parts of the manuscript. HW conducted the experiments, designed the online processing flow and performed behavioral analysis with respect to reaction time and late reaction time, selected the questionnaires and wrote parts of the manuscript. MM conducted the experiments, adjusted the MMI to match the experiments needs and wrote parts of the manuscript. FK contributed to the concept of the MMI, critically discussed the research goals, and revised and improved the manuscript. All authors gave their final approval of the version to be published and agreed to be accountable for all aspects of the work in ensuring that questions related to the accuracy or integrity of any part of the work are appropriately investigated and resolved.

## Funding

This work was funded by research grants from the German Federal Ministry for Economic Affairs and Energy (grant FKZ 50 RA 1011, FKZ 50 RA 1012 and grant FKZ 50 RA 1301).

### Conflict of interest statement

The authors declare that the research was conducted in the absence of any commercial or financial relationships that could be construed as a potential conflict of interest. The reviewer PK and handling Editor declared their shared affiliation, and the handling Editor states that the process nevertheless met the standards of a fair and objective review.

## References

[B1] Allanson J. Fairclough S. (2004). A research agenda for physiological computing. Interact. Comput. 16, 857–878. 10.1016/j.intcom.2004.08.001

[B2] Chang C.-C. Lin C.-J. (2011). LIBSVM: a library for support vector machines. ACM Trans. Intell. Syst. Technol. 2, 27:1–27:27. 10.1145/1961189.1961199

[B3] Cornellă J. Zerbato D. Giona L. Fiorini P. Sequeira V. (2012). Dynamics simulation for the training of teleoperated retrieval of spent nuclear fuel, in ICRA (St. Paul, MN), 5012–5017.

[B4] Devlaminck D. Wyns B. Grosse-Wentrup M. Otte G. Santens P. (2011). Multisubject learning for common spatial patterns in motor-imagery BCI. Comput. Intell. Neurosci. 2011, 1–9. 10.1155/2011/217987 22007194 PMC3191786

[B5] DFKI - RIC (2015). Mars - a Cross-Platform Simulation and Visualization Tool. Available online at: http://rock-simulation.github.io/mars/, last visited on 04.11.2015.

[B6] Duncan-Johnson C. C. Donchin E. (1977). On quantifying surprise: the variation of event-related potentials with subjective probability. Psychophysiology 14, 456–467. 905483 10.1111/j.1469-8986.1977.tb01312.x

[B7] Folgheraiter M. Jordan M. Straube S. Seeland A. Kim S. K. Kirchner E. A. (2012). Measuring the improvement of the interaction comfort of a wearable exoskeleton. Intern. J. Soc. Robot. 4, 285–302. 10.1007/s12369-012-0147-x

[B8] Ghaderi F. Kim S. K. Kirchner E. A. (2014). Effects of eye artifact removal methods on single trial P300 detection, a comparative study. J. Neurosci. Methods 221, 41–47. 10.1016/j.jneumeth.2013.08.025 24056231

[B9] Ghaderi F. Straube S. (2013). An adaptive and efficient spatial filter for event-related potentials, in Signal Processing Conference (EUSIPCO), 2013 Proceedings of the 21st European (Marrakech), 1–5.

[B10] Hart S. G. Staveland L. E. (1988). Development of nasa-tlx (task load index): Results of empirical and theoretical research. Adv. Psychol. 52, 139–183.

[B11] Isreal J. Chesney G. Wickens C. Donchin E. (1980). P300 and tracking difficulty: evidence for multiple resources in dual-task performance. Psychophysiology 17, 259–273. 7384376 10.1111/j.1469-8986.1980.tb00146.x

[B12] Jansen B. Allam A. Kota P. Lachance K. Osho A. Sundaresan K. (2004). An exploratory study of factors affecting single trial P300 detection. IEEE Trans. Biomed. Eng. 51, 975–978. 10.1109/TBME.2004.826684 15188867

[B13] Kahneman D. (1973). Attention and Effort. Englewood Cliffs, NJ: Prentice Hall.

[B14] Kim S. K. Kirchner E. A. (2012). Preliminary results on p300 detection using machine learning when modulating task reaction time, in Proceedings of the 18th Annual Meeting of the Organization for Human Brain Mapping, OHBM-2012 (Beijing).

[B15] Kindermans P.-J. Verschore H. Verstraeten D. Schrauwen B. (2012). A P300 BCI for the masses: prior information enables instant unsupervised spelling, in Advances in Neural Information Processing Systems 25 (NIPS-2012), eds Bartlett P. Pereira F. Burges C. Bottou L. Weinberger K. (Lake Tahoe), 719–727.

[B16] Kirchner E. A. (2014). Embedded Brain Reading. PhD thesis, University of Bremen, Bremen.

[B17] Kirchner E. A. (2015). Intrinsische intentionserkennung in technischen systemen, in GI-Edition: Lecture Notes in Informatics, Ausgezeichnete Informatikdissertationen 2014, ed Hölldobler S. (Bonn: Bonner Köllen Verlag), 121–130.

[B18] Kirchner E. A. Albiez J. Seeland A. Jordan M. Kirchner F. (2013a). Towards assistive robotics for home rehabilitation, in Proceedings of the 6th International Conference on Biomedical Electronics and Devices (BIODEVICES-13), eds Chimeno M. F. Solé-Casals J. Fred A. Gamboa H. (Barcelona: SciTePress), 168–177.

[B19] Kirchner E. A. de Gea Fernandéz J. Kampmann P. Schröer M. Metzen J. H. Kirchner F. (2015). Intuitive interaction with robots - technical approaches and challenges, in Formal Modeling and Verification of Cyber Physical Systems, ed Kühne R. D. U. (Heidelberg: Springer Verlag GmbH), 224–248.

[B20] Kirchner E. A. Drechsler R. (2013). A formal model for embedded brain reading. Indust. Robot 40, 530–540. 10.1108/IR-01-2013-318

[B21] Kirchner E. A. Kim S. K. Straube S. Seeland A. Wöhrle H. Krell M. M.. (2013b). On the applicability of brain reading for predictive human-machine interfaces in robotics. PLoS ONE 8:e81732. 10.1371/journal.pone.0081732 24358125 PMC3864841

[B22] Kirchner E. A. Tabie M. Seeland A. (2014). Multimodal movement prediction - towards an individual assistance of patients. PLoS ONE 9:e85060. 10.1371/journal.pone.0085060 24416341 PMC3885685

[B23] Kirchner E. A. Wöhrle H. Bergatt C. Kim S.-K. Metzen J. H. Feess D. (2010). Towards operator monitoring via brain reading – an EEG-based approach for space applications, in Proceedings of the 10th International Symposium on Artificial Intelligence, Robotics and Automation in Space (Sapporo), 448–455.

[B24] Kok A. (2001). On the utility of P3 amplitude as a measure of processing capacity. Psychophysiology 38, 557–577. 10.1017/S0048577201990559 11352145

[B25] Kramer A. F. Trejo L. J. Humphrey D. (1995). Assessment of mental workload with task-irrelevant auditory probes. Biol. Psychol. 40, 83–100. 7647188 10.1016/0301-0511(95)05108-2

[B26] Krauledat M. Tangermann M. Blankertz B. Müller K.-R. (2008). Towards zero training for brain-computer interfacing. PLoS ONE 3:e2967. 10.1371/journal.pone.0002967 18698427 PMC2500157

[B27] Krell M. M. Straube S. Seeland A. Wöhrle H. Teiwes J. Metzen J. H.. (2013a). pySPACE - a signal processing and classification environment in Python. Front. Neuroinformat. 7:40. 10.3389/fninf.2013.00040 24399965 PMC3871959

[B28] Krell M. M. Tabie M. Wöhrle H. Kirchner E. A. (2013b). Memory and processing efficient formula for moving variance calculation in EEG and EMG signal processing, in Proc. International Congress on Neurotechnology, Electronics and Informatics (NEUROTECHNIX 2013) (Vilamoura: ScitePress), 41–45.

[B29] Kyndt E. Dochy F. Struyven K. Cascallar E. (2011). The direct and indirect effect of motivation for learning on students approaches to learning through the perceptions of workload and task complexity. Higher Educ. Res. Dev. 30, 135–150. 10.1080/07294360.2010.501329

[B30] Li Y. Guan C. Li H. Chin Z. (2008). A self-training semi-supervised SVM algorithm and its application in an EEG-based brain computer interface speller system. Pattern Recogn. Lett. 29, 1285–1294. 10.1016/j.patrec.2008.01.030

[B31] Lotte F. Guan C. (2010). Learning from other subjects helps reducing brain-computer interface calibration time, in Acoustics Speech and Signal Processing (ICASSP), 2010 IEEE International Conference on (Dallas, TX), 614–617.

[B32] Lu S. Guan C. Zhang H. (2009). Unsupervised brain computer interface based on intersubject information and online adaptation. Neural Syst. Rehabilit. Eng. IEEE Trans. 17, 135–145. 10.1109/TNSRE.2009.2015197 19228561

[B33] Mertens R. Polich J. (1997). P300 from a single-stimulus paradigm: passive versus active tasks and stimulus modality. Electroencephalogr. Clin. Neurophysiol. 104, 488–497. 9402891 10.1016/s0168-5597(97)00041-5

[B34] Metzen J. H. Kirchner E. A. (2011). Rapid adaptation of brain reading interfaces based on threshold adjustment, in Proceedings of the 2011 Conference of the German Classification Society (GfKl-2011), 138.

[B35] Polich J. (1990). P300, probability, and interstimulus interval. Pyschophysiology 27, 396–403. 2236442 10.1111/j.1469-8986.1990.tb02333.x

[B36] Polich J. (2007). Updating P300: an integrative theory of P3a and P3b. Clin. Neurophysiol. 118, 2128–2148. 10.1016/j.clinph.2007.04.019 17573239 PMC2715154

[B37] Polich J. Margala C. (1997). P300 and probability: comparison of oddball and single-stimulus paradigms. Intern. J. Psychophysiol. 25, 169–176. 9101341 10.1016/s0167-8760(96)00742-8

[B38] Pope A. T. Bogart E. H. Bartolome D. S. (1995). Biocybernetic system evaluates indices of operator engagement in automated task. Biol. Psychol. 40, 187–195. 7647180 10.1016/0301-0511(95)05116-3

[B39] Prinzel L. J. Freeman F. G. Scerbo M. W. Mikulka P. J. Pope A. T. (2003). Effects of a psychophysiological system for adaptive automation on performance, workload, and the event-related potential p300 component. Human Fact. 45, 601–614. 10.1518/hfes.45.4.601.27092 15055457

[B40] Rivet B. Cecotti H. Perrin M. Maby E. Mattout J. (2011). Adaptive training session for a P300 speller brain–computer interface. J. Physiol. Paris 105, 123–129. 10.1016/j.jphysparis.2011.07.013 21843639

[B41] Rivet B. Souloumiac A. Attina V. Gibert G. (2009). xDAWN algorithm to enhance evoked potentials: application to brain-computer interface. IEEE Trans. Biomed. Eng. 56, 2035–2043. 10.1109/TBME.2009.2012869 19174332

[B42] Rommerman M. Kuhn D. Kirchner F. (2009). Robot design for space missions using evolutionary computation, in Evolutionary Computation, 2009. CEC '09. IEEE Congress on (Trondheim), 2098–2105.

[B43] Samek W. Meinecke F. Müller K.-R. (2013). Transferring subspaces between subjects in brain-computer interfacing. Biomed. Eng. IEEE Trans. 60, 2289–2298. 10.1109/TBME.2013.2253608 23529075

[B44] Schroeders U. Wilhelm O. (2011). Computer usage questionnaire: Structure, correlates, and gender differences. Comput. Hum. Behav. 27, 899–904. 10.1016/j.chb.2010.11.015

[B45] Straube S. Krell M. M. (2014). How to evaluate an agent's behaviour to infrequent events? – reliable performance estimation insensitive to class distribution. Front. Comput. Neurosci. 8:43 10.3389/fncom.2014.00043 PMC3989732 24782751

[B46] Tabie M. Wöhrle H. Kirchner E. A. (2014). Runtime calibration of online eeg based movement prediction using emg signals, in Proceedings of the 7th International Conference on Bio-inspired Systems and Signal Processing (BIOSIGNALS-14) (Angers: ScitePress), 284–288.

[B47] Tueting P. Sutton S. Zubin J. (1970). Quantitative evoked potential correlates of the probability of events. Psychophysiology 7, 385–394. 5510812 10.1111/j.1469-8986.1970.tb01763.x

[B48] Wickens C. D. (1984). Processing resources in attention, in Varieties of Attention, eds Parasuraman R. Davies D. (London; Oxford; Boston, MA; New York, NY; San Diego, CA: Academic Press), 63–101.

[B49] Wickens C. D. (1992). Engineering Psychology and Human Performance (2nd. Edn.) New York, NY: HarperCollins.

[B50] Wickens C. D. (2008). Multiple resources and mental workload. Hum. Fact. 50, 449–455. 10.1518/001872008X288394 18689052

[B51] Wöhrle H. Kirchner E. A. (2014a). Online classifier adaptation for the detection of p300 target recognition processes in a complex teleoperation scenario, in Physiological Computing Systems, volume 8908 of Lecture Notes in Computer Science, da Silva H. P. Holzinger A. Fairclough S. Majoe D. (Berlin; Heidelberg: Springer), 105–118.

[B52] Wöhrle H. Kirchner E. A. (2014b). Online detection of P300 related target recognition processes during a demanding teleoperation task. in Proceedings of the International Conference on Physiological Computing Systems, (PhyCS 2014), (Lissabon: SCITEPRESS Digital Library), 13–19.

[B53] Wöhrle H. Krell M. M. Straube S. Kim S. K. Kirchner E. A. Kirchner F. (2015). An adaptive spatial filter for user-independent single trial detection of event-related potentials. IEEE Trans. Biomed. Eng. 62, 1696–1705. 10.1109/TBME.2015.2402252 25680204

[B54] Wöhrle H. Teiwes J. Kirchner E. A. Kirchner F. (2013a). A framework for high performance embedded signal processing and classification of psychophysiological data in APCBEE Procedia. International Conference on Biomedical Engineering and Technology (ICBET-2013), 4th, May 19-20 (Kopenhagen: Elsevier), 6066.

[B55] Wöhrle H. Teiwes J. Krell M. M. Kirchner E. A. Kirchner F. (2013b). A dataflow-based mobile brain reading system on chip with supervised online calibration, in Proc. International Congress on Neurotechnology, Electronics and Informatics (NEUROTECHNIX 2013) (Vilamoura: ScitePress), 46–53.

[B56] Wöhrle H. Teiwes J. Krell M. M. Seeland A. Kirchner E. A. Kirchner F. (2014a). Reconfigurable dataflow hardware accelerators for machine learning and robotics, in ECML/PKDD-2014 PhD Session Proceedings (Nancy), 129–138.

[B57] Wöhrle H. Teiwes J. Tabie M. Seeland A. Kirchner E. A. Kirchner F. (2014b). Prediction of movements by online analysis of electroencephalogram with dataflow accelerators, in Proc. International Congress on Neurotechnology, Electronics and Informatics (NEUROTECHNIX 2014) (Rome: ScitePress), 31–37.

